# Deposition of dust with active substances in pesticides from treated seeds in adjacent fields during drilling: disentangling the effects of various factors using an 8-year field experiment

**DOI:** 10.1007/s11356-021-15247-w

**Published:** 2021-07-07

**Authors:** André Krahner, Udo Heimbach, Matthias Stähler, Gabriela Bischoff, Jens Pistorius

**Affiliations:** 1 Julius Kühn Institute (JKI)—Federal Research Centre for Cultivated Plants, Institute for Bee Protection, Messeweg 11-12, 38104 Braunschweig, Germany; 2 Julius Kühn Institute (JKI)—Federal Research Centre for Cultivated Plants, Institute for Plant Protection in Field Crops and Grassland, Braunschweig, Germany; 3 Julius Kühn Institute (JKI)—Federal Research Centre for Cultivated Plants, Institute for Ecological Chemistry, Plant Analysis and Stored Product Protection, Berlin, Germany

**Keywords:** Dust drift deposition, Drilling technique, Adjacent field, Seed treatment, Exposure, Non-target organisms (NTA), Responsible Editor: Ester Heath

## Abstract

The side effects from the use of plant protection products and their potential effects on non-target arthropods (NTAs) such as honey bees, other insects within the vegetation layer and epigeic arthropods nowadays receive more attention. However, uncertainties about the factors driving the deposition of active substances (a.s.) into off-crop areas persist, in particular during sowing of treated seeds. Analysing a highly standardised 8-year field experiment, we assessed the importance of various factors potentially affecting dust drift and deposition of a.s., emitted during the sowing process of treated seeds and deposited on fields adjacent to the drilling field, i.e. on the ground, on flowers, and on nonflowering plant parts. Regarding a.s. deposition, the Heubach a.s. value has a predictive capability, which is independent from all other factors taken into account in this study, and can thus be considered as a scenario-independent measure of potential dust deposition. Petri dish samplers, an established standard method for measuring a.s. deposition, were representative of the results from the plant samplers for a given combination of drilling technique and adjacent crop type. Adjacent crop type is likely to impact on a.s. deposition. The present work will enable a more field-realistic exposure assessment for bees and other NTAs.

**Supplementary Information:**

The online version contains supplementary material available at 10.1007/s11356-021-15247-w.

## Introduction

The potential side effects from the use of plant protection products (PPP) on terrestrial non-target organisms (NTAs) such as honey bees and aquatic NTAs represent a key element of ecotoxicological risk assessment. Abrasion of insecticidal seed treatment particles and their dispersal in the environment during sowing has lead to severe effects on bees—massive bee poisoning incidents were reported in various countries (Nuyttens et al. 2013 and references therein). Drilling of treated seeds poses risks for honey bees and may cause acute poisoning of bees (Pistorius et al. 2009) as well as some long term effects on mortality levels, when insecticidal dust particles have been foraged and stored in cells (Pistorius et al. 2015). Likewise, the use of seed treatments may negatively affect bee species richness in field margins (Main et al. 2020).

Dust particles contaminated with active substances (a.s.) may either originate in wind erosion of contaminated soil (Schaafsma et al. 2015), which arguably contributes comparatively little to the overall wind-mediated exposure (Limay-Rios et al. 2016); or they may be abraded from the treated seed (e.g. Greatti et al. 2003; Schnier et al. 2003). Dust particles may deposit on flowering plants in areas adjacent to the treated field (Alix and Lewis 2011), leading to a contamination of nectar, pollen, and morning dew which may be collected actively by bees. Because part of the dust particles (Foqué et al. 2016) fall in the same size classes compared to pollen grains from bee relevant sources, such as mustard and rape (Beug 2015), dust particles may be erroneously collected, transported to the hive and stored in the combs. Moreover, contaminated dust particles may be collected passively by bees (e.g. Tapparo et al. 2012).

One important factor determining the creation and emission of dust is the abrasion potential (abrasiveness) of the seed treatment (Nikolakis et al. 2009; Heimbach et al. 2014). The abrasion potential is influenced by the type of sown crop due to differences in seed morphology. For example, maize seeds generate higher amounts of dust compared to sugar beet, oilseed rape, and barley seeds (Auteri et al. 2017; Heimbach et al. 2014). Moreover, abrasion potential is influenced by the seed-treatment quality (Friessleben et al. 2010). For the sake of comparability between crops, a measure of the abrasion potential should take the crop specific seed rate into account, e.g. by referring abrasion to the area sown (Zwertvaegher et al. 2016). Taking together the amount of dust abrasion per seed, the amount of a.s. within the abraded dust and the crop specific seed rate, the Heubach a.s. value (HVAS) has been proposed as a useful predictor of potential a.s. deposition in the risk assessment for treated seed drilling (Heimbach et al. 2014). However, until now, the field realistic effect of the HVAS on off-crop a.s. deposition has not been assessed systematically.

The emission of dust into adjacent areas is generally influenced by environmental conditions (Nuyttens and Verboven 2015), such as soil properties, weather condition (including wind speed and direction) and the area sown. Moreover, dust drift from treated seeds is influenced by the technical equipment used for drilling (Rautmann et al. 2009), which can be adapted in order to reduce dust emission and/or drift (Biocca et al. 2011; Forster 2012; Friessleben et al. 2010; Manzone et al. 2014; Manzone and Tamagnone 2018; Nikolakis et al. 2009). However, data useful for assessing the influence of the sowing equipment on field-realistic off-crop a.s. deposition are still scarce.

Different approaches for dust drift field measurements have been adopted in the past (Nuyttens et al. 2013). Petri dishes with wet filter paper inlay are frequently used for sampling dust drift in field trials (e.g., Nikolakis et al. 2009; Pochi et al. 2015). Petri dishes are a standard method to assess spray drift (Miller 2003), and this sampling method may represent a realistic scenario to evaluate bee exposure on soil or leaf surface (EFSA 2012). However, few data are available to answer the question how well the amount of a.s. deposited on Petri dishes represents the deposition in above-soil structures relevant for insects, i.e. flowers and nonflowering plant parts adjacent to the drilled field.

Deposition of dust on plants may be plant species-specific due to differences in leaf structure, hairiness, and other factors (EFSA 2012). Moreover, crop-specific vegetation density may impact dust deposition in the adjacent crop, representing a more or less penetrable wind barrier, exercising more or less resistance to the air currents and thus, specifically altering wind velocity and dust deposition pattern. Depending on the focal NTA, exposure might be more relevant with regard to flowering plant parts, as is the case in bees and other flower visiting insects; nonflowering plant parts, as for e.g. herbivores and plant-sucking insects (via honey dew also for honey bees); or soil (bare soil or soil below the vegetation layer), as for epigeic and other ground exposed organisms, such as several bee taxa collecting soil for nest building. Therefore, a comparison of deposition into these different compartments of the adjacent field is highly relevant for the risk assessment of nontarget organisms, but robust data in this regard are still lacking.

Most of the PPP-related research on dust drift in the past years focussed on neonicotinoid insecticides. Seeds treated with neonicotinoid a.s. are drilled in many regions all over the world and used in many different crops, whilst vigorous conclusions about sublethal effects on bees under field-realistic conditions are still lacking (Alkassab and Kirchner 2017). Whilst most applications of these insecticides were banned in Europe, other a.s. used for seed treatments may also be associated with properties harmful to bees (Siviter et al. 2018), and seed treatments remain as a promising mode of application for PPP in the future. Therefore, the exposure of bees as well as that of other insects to a.s. via dust drift deposition is a highly relevant scenario in agricultural landscapes all over the world. The topic of assessing dust abrasion potential, and thereby dust drift potential has relevance beyond bees, neonicotinoids, and other insecticides.

A number of previous studies measuring dust drift from treated seeds during drilling used an indoor approach (e.g. Manzone et al. 2014). Although the indoor approach enables a high degree of standardisation and modelling may allow transferring the results into real world scenarios to some degree (Devarrewaere et al. 2016b), results from field trials are the most realistic way of assessing the importance of different factors affecting the drift of a.s. within dust from treated seed and the corresponding field-realistic exposure of bees and other crop-visiting insects. Here, we report the outcome of a highly standardised 8-year field experiment specifically designed for this purpose. We are using these data to answer the following questions:

1. To what extent does the HVAS determine a.s. deposition in adjacent areas?
2. What impact has the pneumatic drilling technique (air suction or air pressure) on a.s. deposition?
3. How well are ground-level Petri dish samplers reflecting a.s. deposition in the adjacent crop?
4. Has the presence of an adjacent vegetation layer an impact on the amount of a.s. deposition, and does a.s. deposition differ between flowers and nonflowering plant parts as well as between crop types?

## Material and methods

### Study site and design

**Fig. 1.**
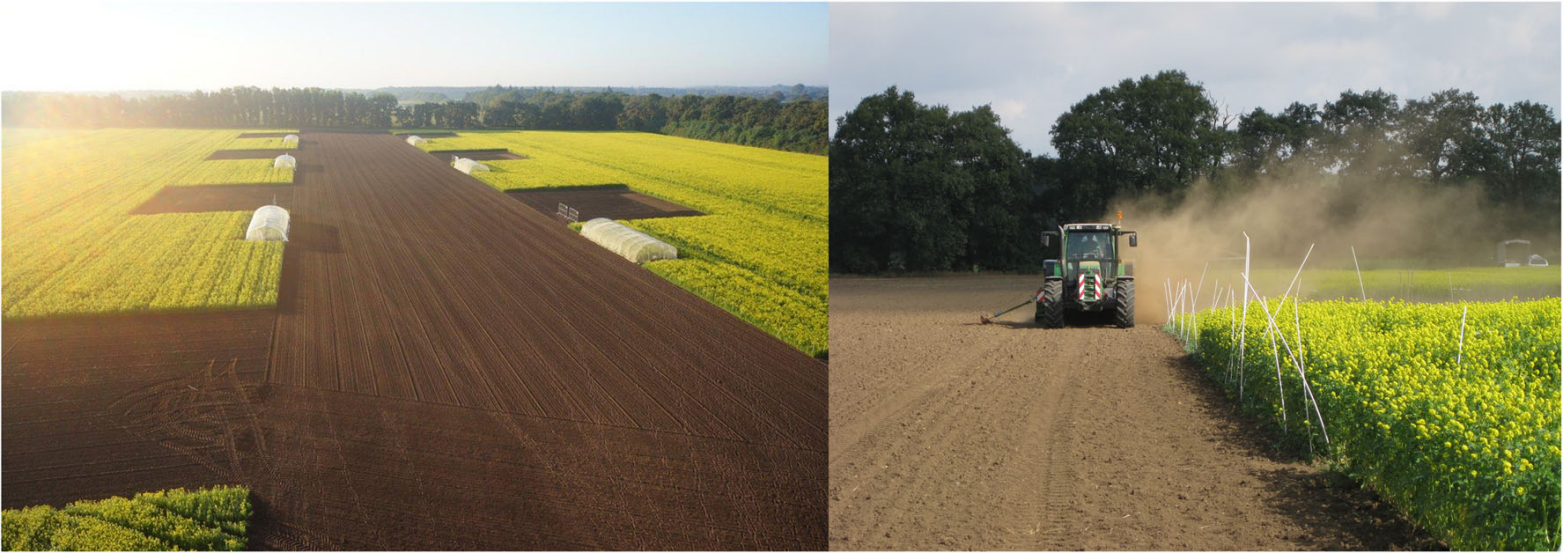
Exemplary study site (left), showing the alternating blocks with flowering crop and bare soil adjacent to the drilling area (centre strip), and drilling of the treated seed with soil dust visible (right). Photos: © Institute for Plant Protection in Field Crops and Grassland, Julius Kühn Institute

Data were aggregated over 13 standardised field trials conducted in the area around Braunschweig, Germany, over 8 years (Table 1). In each trial, treated seeds (maize, rape, barley or wheat) were drilled into an area (45–51 m wide, 240–433 m long) surrounded by an established adjacent crop (rape or mustard; Fig. 1). The drilled area had been cut some days before drilling and was oriented in a way that the predominant wind direction was nearly perpendicular to the centreline of the area. Both along the upwind and downwind side 3 blocks (30 m wide, 30–40 m long) of bare soil were established by removing the existing crop, alternating with 3 still vegetated plots (30 m wide, 50–60 m long). Wind conditions were measured using a weather station (Driesen & Kern MMC). During drilling, mean wind speed was between 1.31 and 3.73 m/s (average 2.38 m/s). Deviation of the wind direction from 90° with regard to the exposed field during drilling is shown in the windrose plots in Fig. S11.

**Table 1:** Overview of the experimental settings of the field trials, including neonicotinoid active substances and the corresponding plant protection products used for seed treatment

Trial (Code)	Year	PPP^1^	a.s.^2^	Drilled crop (cultivar)	Adjacent crop	Drilling method^3^	HVAS [g/ha]^4^
1008	2010	Poncho®	clothianidin	maize (Athletico)	rape	air suction	0.09074
1115	2011	Poncho®	clothianidin	maize (PR36PB5)	rape	air suction	0.08595
1122	2011	Elado®	clothianidin	rape (MaPlus)	mustard	air pressure	0.00342
1205	2012	Poncho Pro®	clothianidin	maize (11MA00581)	rape	air suction	0.04165
1224	2012	Gaucho® FS 600	imidacloprid	barley (LOMERIT)	mustard	air pressure	0.08588
1303	2013	Cruiser®	thiamethoxam	maize (Silvano)	rape	air suction	0.07876
1315	2013	Elado®	clothianidin	rape (Compass)	mustard	air pressure	0.00869
1405	2014	Elado®	clothianidin	rape (NK Technik)	rape	air pressure	0.00464
1411	2014	Elado®	clothianidin	rape (NK Linus)	mustard	air pressure	0.00089
1505	2015	Elado®	clothianidin	rape (NK Sherpa)	rape	air pressure	0.00067
1636	2016	Gaucho® FS 350	imidacloprid	summer wheat (Collada)	rape	air pressure	0.07418
1647	2016	Gaucho® FS 350	imidacloprid	winter wheat (Rockefeller)	rape	air pressure	0.02330
1708	2017	Gaucho® FS 350	imidacloprid	summer wheat (Namib)	rape	air pressure	0.02888

^1^Plant protection product; ^2^neonicotinoid active substance; ^3^air suction with deflectors directed to the soil; ^4^Heubach value expressed as g active substance per ha (rounded to 5 digits)

For drilling, commercially available batches of treated seed were used, reflecting seed treatment qualities usual at the time of the trials. In this way, a broad range of seed treatment qualities was tested, reflecting the continuous improvement of abrasiveness over the years. Seed treatments varied amongst trials and included the products Poncho®, Poncho Pro® and Cruiser® (maize seed), Elado® (rape seed) and Gaucho® (barley and wheat seed).

Petri dishes (surface area: 143 cm^2^, diameter: 13.5 cm, depth: 2 cm) with filter collectors (filter paper; diameter: 12.5 cm; grade 602 H, Fig. S1) were prepared in the laboratory and installed before the drilling event in the adjacent crop as well as in the blocks with bare soil, in distances of 0, 1, 3, and 5 m from the edge of the drilling area, on the wind exposed side. In total, 3 blocks with bare soil and with established adjacent crop, respectively, were sampled, using 3–4 Petri dishes per distance and block (with the exception of the first trial, in which 9–15 Petri dishes were used per block and distance in the blocks with bare soil in order to assess the appropriate number of repeated measurements).

Before drilling, the adjacent crop was wetted using a glycerol-water solution, in order to create a worst-case situation with regard to adhesion of dust particles onto plant parts. Standard spraying equipment was used for glycerol application, the water input being raised to 800 L/ha to allow a better wetting of all plant parts but with still no drops falling off. Petri dishes were opened immediately (24.3 ± 12.3 min; mean ± standard deviation, SD) before drilling. For drilling two different types of machinery were used in different trials: a Kverneland Accord Optima NT e-drive for drilling maize seed (air suction system; exhaust air directed to the soil) or an Amazone ADP Spezial for drilling rape, wheat, and barley seed (air pressure system). Drilling was completed within about one hour (59.0 ± 10.5 min; mean ± SD).

At the same distances as Petri dishes, plant samples were taken from the vegetated blocks of the wind exposed side of the drilling area. In general, 4 samples were taken per distance and block by collecting plant material from a square area of 0.0961 m^2^ (0.31 m * 0.31 m). Inflorescences were collected separately from the rest of the plant, the latter being cut at about 10 cm above the ground. Exposition of Petri dishes was terminated by closing Petri dishes with lids within about 5 min (4.6 ± 4.2 min; mean ± SD) after drilling and plant samples were collected within approximately 2 h after the drilling event (start of plant material collection 4.9 ± 3.2 min after drilling; duration of plant material collection 114.6 ± 23.7 min; mean ± SD). Both Petri dishes and plant samples were deep-frozen immediately after delivery to the analytical laboratory. In order to assess the background residue level in the adjacent field and potential drift from outside the experiment, 4–8 Petri dishes were exposed during drilling and, in most trials, 3 plant samples were taken as described above at 3 m distance from the drilled field in upwind direction with regard to the drilled field.

The Heubach value, giving the amount of dust abraded per seed under standardised conditions, was measured using a Heubach-Dustmeter (Type I, Heubach DUSTMETER GmbH, Oberalm; for a detailed methodology refer to Heimbach 2008). The Heubach a.s. value (HVAS) was calculated by multiplication of (1) the amount of dust per seed grains or seed mass (as determined using the Heubach-Dustmeter), (2) the amount of a.s. per mass of dust (as determined within the dust fraction from the Heubach-Dustmeter through chemical residue analysis; see the “Chemical residue analysis” section below for determination), and (3) the crop specific seed rate in terms of seed grains or seed mass sown per ha.

### Chemical residue analysis

LC-MS/MS was used to quantify and identify the various target substances from the neonicotinoid group, i.e. the active substance applied with the seed treatment. Analysed samples included Petri dishes with filter collectors, plant samples, and filters from Heubach a.s. tests. The system used was an UltiMate 3000 HPLC system (Dionex, since 2011, Thermo Fisher Scientific, Sunnyvale, CA, USA) coupled to a QTRAP 5500 triple quadrupole mass spectrometer (SCIEX, Framingham, MA, USA) equipped with an electrospray ionisation (ESI) source.

For quantification, the internal standard method was used with an appropriate isotope-labelled internal standard for each target analyte and matrix-matched calibration standards. This method minimises the matrix effect and allows accurate quantification. In the case of sample dilution, sample extracts were quantified with isotope-labelled standards in solvent. Deuterated surrogate standards were added to each sample for internal monitoring of analytical quality.

The methods were validated via recovery studies by analysing a series of spiked replicate samples from Petri dishes and plant parts and determining the recovery rates of the target analytes (see supplement, S1). Limits of detection (LOD) and limits of quantification (LOQ) were determined experimentally from the recovery studies where possible and additionally estimated from the signal-to-noise ratios of the matrix standards. Two multiple reaction monitoring (MRM) transitions were used to confirm the identity of each analyte and to determine the respective LOD and LOQ. This approach was sufficient, because the target compounds were known and only one was analysed at a time. The LOQ values of all a.s. were determined at 0.07 μg/m^2^ for the Petri dishes with filter collectors and at 0.10 μg/m^2^ for the plant parts (flowers or stems and leaves). The corresponding LODs of all a.s. and matrices were calculated from the lowest concentration level of the calibration curves and were one tenth below the LOQ. Further details regarding the chemical residue analysis, including information about sample preparation, extraction procedures, and method validation, are presented in the supplement (S1).

### Statistical analyses

In order to enable the adoption of a gamma-distribution for GLMMs, a value of 0.0001 μg/m^2^ and 0.0001 μ/kg, respectively, was assigned to samples below the limit of detection (LOD) prior to the analysis, which is two orders of magnitude lower than the minimum measured residue in the datasets. Since exact values measured between the LOD and the limit of quantification (LOQ) were not available, samples > LOD and < LOQ were set to the LOQ, following the approach proposed by EFSA (2013).

Three datasets were analysed separately: a multiple-sampler, a plant material-only, and a Petri dish-only dataset. The first dataset consisted of residue measurements sampled in different matrices described above, but only within the adjacent crop (1390 data points). The second dataset was derived from the first dataset and restricted to plant samples (flowers, stems, and leaves), for which meaningful measures of μg a.s. per kg sampling material were available (833 data points). The third dataset consisted of residue measurements in Petri dishes only, but within the adjacent crop as well as in the adjacent bare soil (1218 data points). The amount of residues in terms of a.s. per area (μg/m^2^) or a.s. per mass of sampling material (μg/kg) was analysed by means of GLMMs with block nested in trial number as random factors, a log-link function, and a gamma error distribution. Full random-intercept models were created containing distance to the drilled field (“Distance”; 0, 1, 3, and 5m; as a categorical predictor), Heubach value expressed as g a.s./ha (HVAS; log-transformed continuous predictor) and sampler (Petri dish, flowering and nonflowering plant parts) as fixed effects. As trials did not cover all combinations of drilling method (air pressure system or air suction system with deflectors directed to the soil) and crop type adjacent the drilled field (winter oilseed rape or mustard), these two variables were introduced as one combined fixed effect “drilling method-adjacent crop (DMAC)” in the models (air pressure and mustard, air pressure and rape, air suction and rape). Except for the effect of the different samplers (multi-sampler dataset used) and for effects on the mass-related amount of a.s. deposited on plants (plants-only dataset used), analysis based on the Petri dish-only dataset, and only for the analysis of this dataset, HVAS was introduced in the models.

For selection of most parsimonious models, Akaike Information Criterion (AICc) was used, comparing the full models to all possible reduced models, holding the random effect structure constant. Models were validated through visual inspection of residual plots. The variability explained by the selected models (fixed effects and entire model) was determined by calculating the marginal (R^2^_m_) and conditional coefficient of determination (R^2^_c_), respectively, based on Nakagawa et al. (2017). The Tukey method was used for P value correction for post-hoc pairwise comparisons.

For the comparison of residues sampled in different samplers (Petri dishes and plant matrices), only data from trials were used where all types of samplers were situated in the same blocks and receiving the same glycerol treatment. Samples yielding unreliable measurements due to documented improper handling during sample preparation and analysis were removed prior to the analysis.

All analyses were conducted in the R environment version 3.6.3 (R Core Team 2020). GLMMs were fit using function ‘glmer’ from the ‘lme4’ package version 1.1-21 (Bates et al. 2015). For automated model selection the ‘dredge’ function from the ‘MuMIn’ package version 1.42.1 (Barton 2018) was used. Function ‘r.squaredGLMM’ from the ‘lmtest’ package version 0.9-37 (Zeileis and Hothorn 2002) was used to calculate coefficients of determination. Graphics were created using the ‘ggplot’ function from the ‘ggplot2’ package version 3.1.0 (Wickham 2016). For post hoc tests function ‘emmeans’ from the ‘emmeans’ package 1.4.7 was used (Lenth 2020). Statistical differences were determined at α = 0.05.

## Results

The original data have been published for open access (Krahner et al. 2021). For the multisampler dataset, the selected model contained the Distance*DMAC and the DMAC*Sampler interactions. For the plant samples-only dataset, a model containing Distance*Sampler and DMAC*Sampler interactions was selected. For the Petri dish-only dataset, the selected model contained HVAS and the Distance*DMAC interaction. A major part of the variation within the dataset was explained by the models (multisampler dataset: marginal pseudo-R^2^ = 51.8%; conditional pseudo-R^2^ = 69.0%; plant material-only dataset: marginal pseudo-R^2^ = 73.2%; conditional pseudo-R^2^ = 84.0%; Petri dish-only dataset: marginal pseudo-R^2^ = 60.0%; conditional pseudo-R^2^ = 77.3%). Please refer to the supplementary information for detailed results from the GLMMs (Table S7-S9) and post-hoc test statistics on all pairwise comparisons (Tables S10-S17). Residue values measured in samples taken in the upwind direction with regard to the drilling area in most cases did not exceed the LOQ or only on a small scale relative to the LOQ (Fig. S12). As such, these measurements were not considered further in the analysis.

### Heubach a.s. value impacts on deposited a.s. residue value

**Fig. 2.**
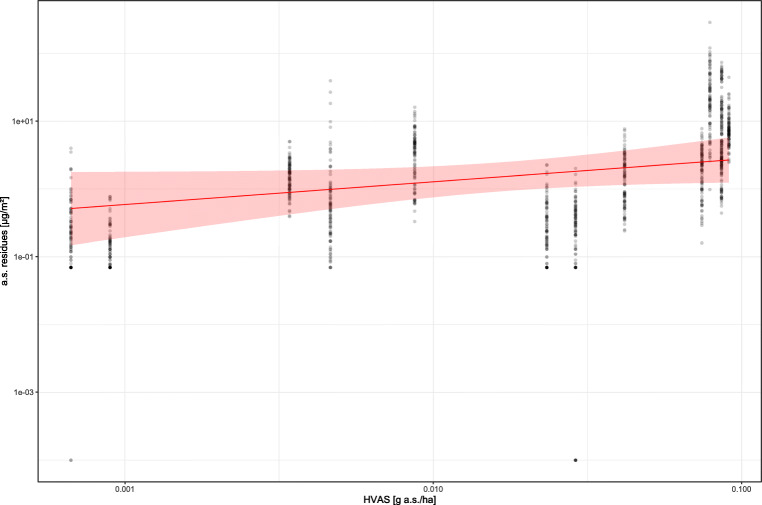
Effect of HVAS of drilled seed on measured residues from Petri dishes in the adjacent field (*n* = 1218 observations). Red: model fit (mean and 95% CI). Black: measured residue data. Note that the results from two trials (HVAS of 0.0859 and 0.0860 g a.s./ha) are virtually indistinguishable in this figure

As expected, the Heubach a.s. value has a positive effect on the deposited a.s. residue values (coefficient: 0.3356, standard error: 0.1793; Fig. 2; Table S18). On average, duplication of the HVAS leads to a 26.2% increase in measured residue values, and a 10fold increase in the HVAS results in a duplication (216.6% increase) in measured residue values.

### Effect of the sampler

**Fig. 3.**
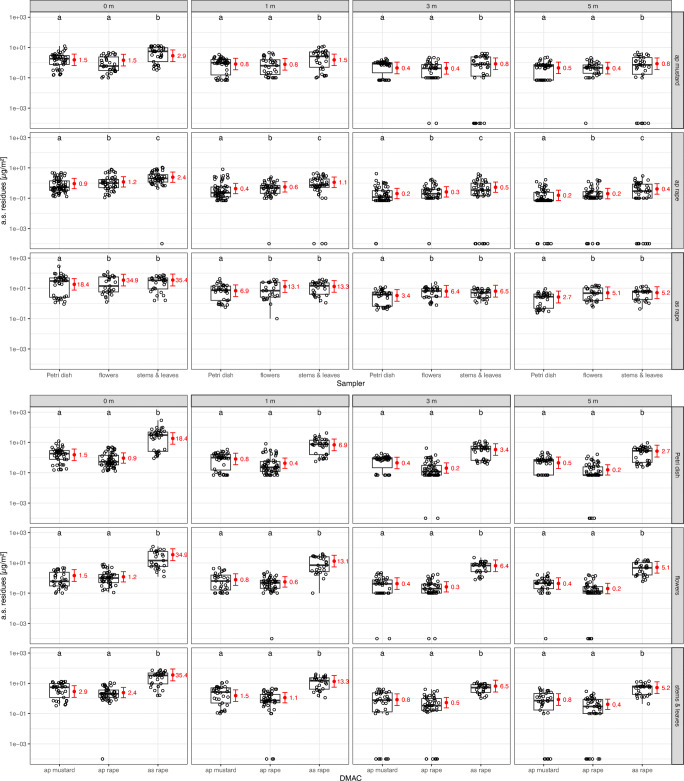
Effect plots for the SAMP*DMAC and distance*DMAC interactions at different distances to the drilled field (*n* = 1390 observations). Red: model fit (mean and 95% CI). Black: measured residue data. ap/as = air pressure/suction drilling. Different letters mark significant differences (α = 0.05)

The sampler has an effect on measured residues in terms of μg/m^2^, interacting with the DMAC effect. In general, stem and leaf samples contained significantly higher residues compared to Petri dishes (Fig. 3; Tables S8 and S14), the former on average being 1.88 (air pressure, mustard), 2.62 (air pressure, rape), and 1.93 (air suction, rape) times higher than the latter. For air pressure drilling, stem and leaf residues were significantly higher compared to flower residues as well (Fig. 3, Tables S8 and S14), the former on average being 1.96 (mustard) and 2.03 (rape) times higher than the latter. No difference between stem and leaf residues and flower residues was observed for air suction drilling. In rape, flower samples contained significantly higher residues compared to Petri dishes (Fig. 3, Tables S8 and S14), the former on average being 1.29 (air pressure) and 1.90 (air suction) times higher than the latter. In all samplers, residues from air suction drilling in rape were significantly higher compared to air pressure drilling, whilst no significant differences were found between air pressure drilling in mustard and rape (Fig. 3, Tables S8 and S13).

### Mass-related amount of a.s. deposited on flowers and nonflowering plant parts in the adjacent crop

**Fig. 4.**
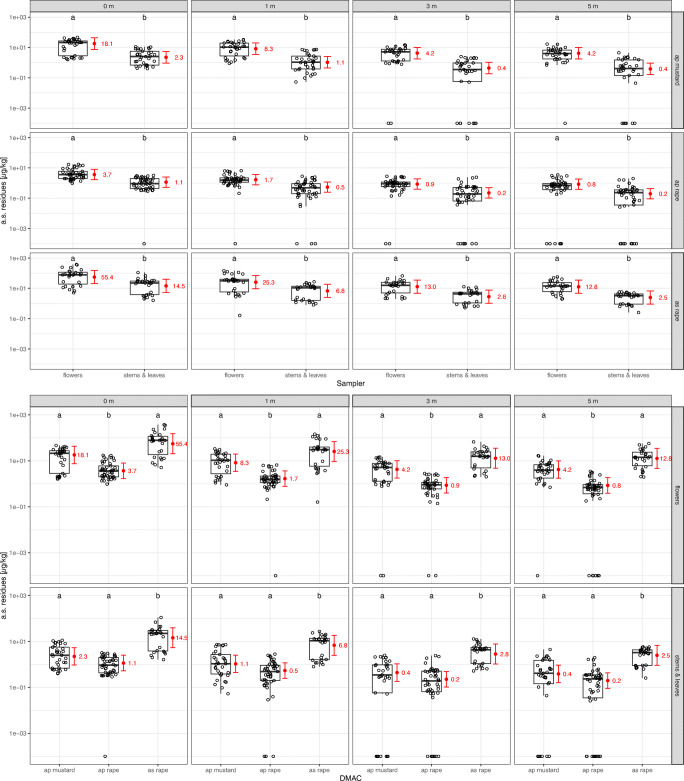
Effect plots for the sampler*DMAC and sampler*distance interactions (*n* = 833 observations). Red: model fit (mean and 95% CI). Black: measured residue data. ap/as = air pressure/suction drilling. Different letters mark significant differences between samplers (α = 0.05)

For some pair-wise comparisons, results obtained for the mass-related amount of a.s. deposited on plants (Fig. 4) differed markedly from the area-related approach (Fig. 3). With regard to the mass-related amount of a.s. deposition, flowers and nonflowering plant parts are significantly different. Irrespective of DMAC and distance to the seeding area, the amount of a.s. per kg plant material is significantly higher in flowers compared to stems and leaves of the adjacent crop (Fig. 4, Tables S9 and S17). Depending on the distance to the seeding area, the difference ranged from about 7.82–10.80 fold (air pressure, mustard), 3.11–4.29 fold (air pressure, rape), and 3.71–5.13 fold (air suction, rape), respectively.

For the mass-related amount of a.s. deposition on stems and leaves, no significant differences were found between mustard and rape (Fig. 4, Tables S9 and S16; air pressure drilling), whilst air suction drilling in rape led to significantly higher a.s. deposition than air pressure drilling at all distances from the drilled field. The a.s. deposition was on average 12.63 times higher in air suction drilling compared to air pressure drilling. Mass-related amount of a.s. deposition on flowers was also higher following air suction drilling compared to air pressure drilling (Fig. 4, Tables S9 and S16; on average 15.08 times higher), whilst significantly higher amounts of a.s. deposited on mustard compared to rape flowers were observed (on average 4.93 times higher).Thus, comparing the a.s. amount related to the mass of plant material leads to contrasting results compared to the area-related a.s. amount.

### Effect of distance on a.s. deposition on the ground

**Fig. 5.**
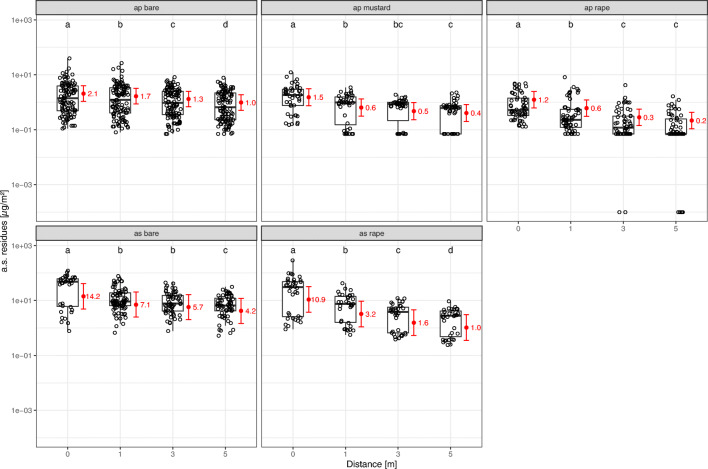
Effect plots for the distance*DMAC interaction (*n* = 1218 observations). Red: model fit (mean and 95% CI). Black: measured residue data. ap/as = air pressure/suction drilling. bare = bare soil. Different letters mark significant differences between distance levels (α = 0.05)

As expected, distance to the drilled field has an effect on the measured residues. An interaction between this effect and the effect of DMAC was observed (Fig. 5). In general, measured residues decrease with increasing distance at all tested combinations of drilling technique and adjacent crop. Although not all (but most) pairwise comparisons yield significant differences between distance levels (Tables S7 and S10), a marked and monotonic trend of decreasing residue measurements with increasing distance was observed also in all cases where differences were not statistically significant (Fig. 5). For air pressure drilling, residue decrease from 0 to 5m was strongest for measurements in rape (Table S7; 83% decrease in fitted means), followed by measurements in mustard (73%) and on bare soil (53%). For air suction drilling, residue decrease from 0 to 5m was stronger for measurements in rape (90% decrease) compared to measurements on bare soil (71%).

### Effect of crop type on a.s. deposition on the ground

**Fig. 6.**
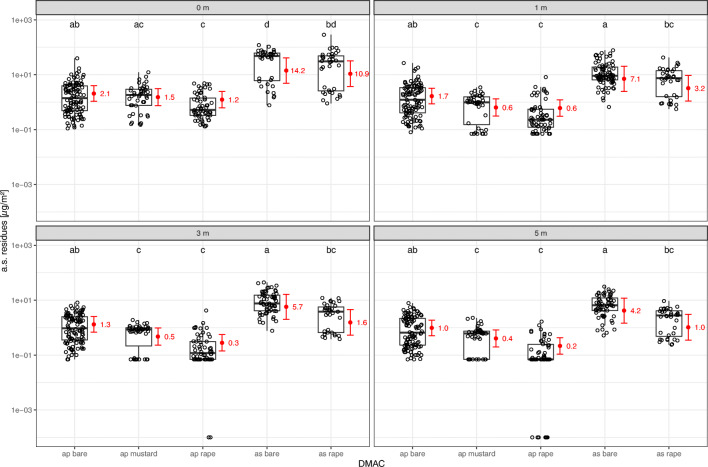
Effect plots for the distance*DMAC interaction (*n* = 1218 observations). Red: model fit (mean and 95% CI). Black: measured residue data. ap/as = air pressure/suction drilling. bare = bare soil. Different letters mark significant differences between DMAC levels (α = 0.05)

Adjacent crop type has an effect on measured a.s. residue values, interacting with the effect of distance to the field (Fig. 6). For air pressure drilling, residues in rape on soil were significantly lower compared to bare soil at all distances (Tables S7 and S11), mean values for the latter being 1.67 (0 m), 2.72 (1 m), 4.66 (3 m), and 4.52 (5 m) times higher than the former. With the exception for measurements at 0 m, residues in mustard on soil were also lower compared to bare soil, with mean residue values in bare soil being 2.58 (1 m), 2.77 (3 m), and 2.42 (5 m) times higher compared to the mustard crop. Whilst a trend towards lower residue values in rape compared to mustard was observed, there was no significant difference between the two crops at any distance (Tables S7 and S11). For air suction drilling, residues in soil below rape were significantly lower compared to bare soil (Tables S7 and S11), except for measurements at 0 m, mean values for the latter being 2.19 (1 m), 3.69 (3 m), and 4.03 (5 m) times higher than the former.

### Effect of drilling technique on a.s. deposition on the ground

Furthermore, an effect of drilling technique could be observed to some degree. For both residues in rape and bare soil, higher residue values were observed for air suction compared to air pressure drilling (Fig. 6). However, these differences were only significant at a distance of 0 m (Fig. 6, Tables S7 and S11). Mean residue values measured at 0 m following air suction drilling were 6.85 (bare soil) and 8.76 (rape) times higher compared to air pressure drilling.

## Discussion

Residue values from samples in upwind direction with regard to the drilled experimental field exceeded the LOQ in some of the trials (Fig. S12). This may be a result from dust drift from outside the experimental field, due to changes in wind direction during drilling or, in the case of plant samples, due to uptake from the soil. As most of these measurements are relatively low compared to the LOQ and the measurements in the downwind direction, measurements in the upwind direction were not considered further in the discussion.

### The Heubach a.s. value determines a.s. deposition to some extent

As expected, the Heubach a.s. value has a positive effect on the deposited a.s. residue values (Fig. 2). The rate of change in deposited residues per unit change in Heubach a.s. value is in the same order of magnitude as the change in ground deposited residues associated with a change in Heubach value observed by Friessleben et al. (2010). Our results, obtained from field-realistic experiments, present a solid ground for the adoption of seed treatment quality parameters by means of the Heubach test in combination with active substance analysis and seed rate in the exposure assessment of insects in crops and bare fields adjacent to the drilled field. Foque et al. (2017) argue in favour of other methods of measuring abrasion potential than the Heubach test, as the latter does not take the larger-size fraction of particles into account, which are probably more relevant for nontarget areas close to the drilling field edge. The present results, however, indicate a relevance of Heubach test results in the exposure assessment of dust drift for areas of up to 5 m from the drilling field edge, i.e. the maximum distance observed by Devarrewaere et al. (2016a) for the movement of particles larger than 500 μm. Moreover, the high amounts of variation already explained by the models presented here let us argue that the potential gain of explained variation through consideration of larger-size particles is rather low. In fact, as a consequence of the improvements in seed cleaning quality in the last years, the larger-size fraction of dust particles can be considered irrelevant in the exposure assessment today.

### Difference between air suction and air pressure drilling technique

The results from the present study indicate that seed drilling methodology may be a factor in dust drift mitigation measures. Biocca et al. (2017a) describe technical devices as generally affordable solutions for dust drift mitigation in precision pneumatic seed drills, with deflectors being the devices most easily retrofitted to existing machinery. Biocca et al. (2011) reported an overall average reduction of a.s. residues of about 58.7%, and Friessleben et al. (2010) observed still greater reduction levels. Even though the seed drilling device based on air suction technique was equipped with a deflector directed to the soil, it still resulted in higher amounts of deposited residues compared to the air-pressure drilling device, with significant differences at least on the edge of the drilling field. This is in contradiction to Nikolakis et al. (2009), who found that, with regard to dust drift reduction, all modified vacuum-pneumatic drilling machines performed in a way comparable to a drilling machine operated with compressed air.

The results from the present comparison of air suction and air pressure technique have to be treated with caution. First, irrespective of the principle mechanism of the drilling equipment (i.e. air suction or air pressure), some differences exist between the various drilling devices on the market with regard to dust drift (Rautmann et al. 2009). Second, in the present study, maize seed was drilled using air suction drilling equipment, whilst air pressure technique was associated with drilling rape, barley, and wheat seed. This lack of factor combinations (e.g., no drilling of maize seeds using air pressure methodology) has to be kept in mind when interpreting the present results, although most of the effect of the seeded crop on a.s. deposition is probably taken into account by means of the HVAS in the present model. Moreover, whilst the upper end of the HVAS spectrum covered in the present study has a balance of trials using air pressure and air suction technique, the lower end of the spectrum is biased towards trials using air pressure drilling. Therefore, the present comparison of air suction and air pressure drilling may be regarded as indicative. In order to derive stronger conclusions with regard to the role of drilling technique in a.s. deposition, further trials would be necessary with both drilling techniques being equally spread across the observed HVAS spectrum. Finally, it has to be noted that meanwhile further progress has been made with regard to the development of technical solutions (e.g. Biocca et al. 2017b).

### Ground-level Petri dish samplers reflect a.s. deposition in the adjacent crop (flowering and nonflowering plant parts)

The employed dust sampler is an important factor determining the amount of residues sampled, and the effect of the dust sampler on the amount of sampled residues is depending on the combination of adjacent crop and drilling technique. This has to be taken into account when translating outcomes of residue trials using Petri dishes to residue amounts in dust samplers arguably more relevant for bees and other insects in the vegetation layer (e.g. flowers, stems, and leaves). Notwithstanding the often observed significant difference between Petri dishes and plant samplers, this difference is relatively unaffected by the distance to the drilled field, within a given combination of adjacent crop and drilling technique. Thus, for a given setting the results from Petri dish samplers can be regarded as representative of the results from the plant samplers. From our results, over all combinations of DMAC and distance to the drilled field, a.s. amounts per area were on average no more than about 2.5 times higher in stems and leaves and no more than about 1.9 times higher in flowers compared to a.s. amounts per area in Petri dishes in the adjacent crop. These relations may be useful in extrapolating from results from Petri dishes in other studies to simulated vegetation samplers. This approach would still further improve our knowledge about a.s. exposure in crops adjacent to the drilled field, even though this exposure would not have been assessed directly. Finally, it has to be noted that ground based Petri dishes by design yield information about potential exposure related to the area observed. This area-related approach yields results different from mass-related deposition, which might be a more suitable approach for the exposure of insect organisms higher up in the vegetation structure (see below).

### The adjacent vegetation layer has an impact on the amount of a.s. deposition

In general, the present results obtained from plant samplers have to be regarded as worst-case scenarios, since the vegetation had been wetted before the drilling event, resulting in an increased adherence of dust particles containing a.s. to the plant surface. This has to be kept in mind when interpreting the results and deriving recommendations for the ecotoxicological risk assessment.

#### Distance matters

As expected, residue levels decrease with increasing distance to the drilling field edge (Fig. 5). The present results indicate a sharp decrease in dust drift residue deposition over the first few metres from the drilled field, with the minimum reduction observed at 5 m being more than 50% compared to the edge of the drilled field. This sharp decrease over close distances to the drilled field is in agreement with field-trial results from Krupke et al. (2017), who observed residue deposition over a much longer distance of 100 m from the drilled field, and Nikolakis et al. (2009), who observed a distance of up to 50 m. A posteriori, these results justify the focus of the present study on a.s. deposition close to the drilled field.

Earlier modelling results indicated a peak deposition 4.2 m away from the drilling field edge (Devarrewaere et al. 2018). In contrast to this result, average peak deposition here was measured directly at the field edge, and deposition values decreased continuously with growing distance. Compared to the scenario modelled by Devarrewaere et al. (2018), the drilling fields in the present study are much narrower. However, as the working width in the present study is also much smaller compared to Devarrewaere et al. (2018), the number of passages here (15–19 passages) is in the same order of magnitude compared to the scenario in Devarrewaere et al. (2018) (13 passages). We cannot exclude different experimental settings as a reason explaining the different dust deposition pattern between the present study and the study by Devarrewaere et al. (2018). However, we would advocate in favour of realistic field trials as represented in the present study, which could then help further improving modelling approaches to the exposure assessment.

#### Relevance of the adjacent crop for epigeic and other ground exposed NTAs

The existence of a crop adjacent to the drilling field leads to a significant reduction in a.s. residue deposition on the ground. This holds true for all distances from the drilled field and for both crop types compared to the adjacent field without any vegetation, except for the edge between drilled area and adjacent field (Fig. 6). Therefore, in the exposure assessment for epigeic and other ground exposed NTAs, such as bees collecting soil for nest building, the presence of a vegetation structure has to be taken into account. Epigeic or ground exposed NTAs are likely to be significantly less exposed to deposited a.s. in adjacent crops compared to adjacent bare fields. With a minimum observed increase of more than two-fold in bare fields compared to crops between 1 and 5 m from the drilled field, this difference is considered biologically relevant. Generally, the results match the observations for spray drift: Koch et al. (2003) emphasised the impact of canopy roughness and filtering effects on spray drift and retention processes, altering the deposition gradient in vegetation structures compared to bare grounds.

Moreover, with regard to ground deposition of a.s., there are indications that exposure to epigeic or ground exposed NTAs is depending on the type of the adjacent crop. Reduction of ground deposition over the first 5 m from the drilled field is higher in rape than in mustard crops, and least high in bare fields (Fig. 5). Although not significant, a coherent trend towards lower amounts of a.s. on the ground was observed in rape compared to mustard (Fig. 6). Further, compared to bare fields, there is a clear pattern of higher reduction with increasing distance in rape, whilst this reduction is more or less constant over the observed distances in mustard (Fig. 6).

#### Relevance of the adjacent crop for NTAs in the nonflowering vegetation layer

NTAs potentially exposed to a.s. depositions in the nonflowering vegetation layer cover a wide range of taxa, ranging from phytophagous organisms with a continuous presence over long time periods to taxa only sporadically present, such as bees collecting nest building material, guttation water or honey dew. No significant difference is observed in a.s. deposition on stems and leaves between mustard and rape crops. This holds true for the area-related (Fig. 3) as well as the mass-related amount of a.s. residues (Fig. 4). However, a general trend towards higher a.s. residues in mustard is observed compared to rape, which is more pronounced considering the mass-related (Fig. 4) compared to the area-related residue amount (Fig. 3). Higher mass-related residues in mustard may be explained by less plant weight per m^2^ collected in this crop compared to oilseed rape. From these results adjacent crop type does not emerge as a primary driver of deposited a.s. exposure to NTAs associated with nonflowering plant parts, if the total plant biomass is taken into account. Rather, using the information from mustard crops in the exposure assessment appears as a conservative (but not over-conservative) approach, enveloping the potential exposure in rape crops.

#### Relevance of the adjacent crop for NTAs in the flowering vegetation stratum

The flowering stratum plays a pivotal role in the exposure assessment for NTAs foraging for nectar and pollen, such as bees, as well as other flower visiting NTAs. For example, pollen from nontarget plants can be a major route of exposure to neonicotinoids for honey bees (Botías et al. 2015). With regard to mass-related a.s. residues, flowers in mustard crops have higher a.s. depositions compared to flowers in rape crops (Fig. 4). Again here the total biomass collected in the two different crops has to be considered. This difference was on average almost 5-fold and is considered biologically relevant. In contrast, no significant differences between the two observed crop types are observed in terms of area-related a.s. residues. However, the results related to plant mass are considered more relevant for the exposure assessment of NTAs for two reasons. First, flower density is expected to be positively correlated with flower visitation rate by insects. Flower density is taken into account by the mass-related and not by the area-related approach. Second, the mass of flowering plant parts is expected to be positively correlated with the amount of nectar and pollen within the same crop. Therefore, mass-related a.s. deposition is likely to represent exposure to nectar and pollen collecting NTAs better than the area-related approach, resembling a proxy for the perceived environmental concentration. Thus, the results of the present study underscore the consideration of the adjacent crop type in the exposure assessment for NTAs in the flowering vegetation stratum.

Because mustard and rape flowers are morphologically similar, it seems unlikely that different scavenger characteristics explain a major part of the differences in mass-related residues in flowers; i.e. mustard flowers are unlikely to scavenge relatively higher amounts of dust particles from the air current compared to rape flowers due to flower morphology. Another explanation seems more likely: a crop-specific function of the vegetation as a wind barrier, which for spray drift has been reviewed by Felsot et al. (2010).

The adjacent crop may govern dust deposition via vegetation density and the resulting deviation and/or deceleration of dust bearing air currents. First, high-density vegetation structures are likely to represent a nearly impenetrable wind barrier, such that air currents are directed above the adjacent crop, with higher wind velocities, longer distances of dust deposition and lower deposition close to drilled field. Second, low-density vegetation structures are likely to represent a low resistance to the wind compared to higher-density vegetation structures, such that air currents are passing through the adjacent crop at higher speed, leading to longer distances of dust deposition, and lower deposition close to the drilled field. For spray drift, these two scenarios have been proposed by Ucar and Hall (2001), and de Schampheleire et al. (2009) observed corresponding results in field trials with regard to spray drift. Finally, intermediate-density vegetation structures are probably penetrable to the wind, but slowing down the air currents and thus, leading to a higher dust deposition closer to the drilled field. For spray drift, de Schampheleire et al. (2009) and Vieira et al. (2018) observed corresponding results in field trials with *Fagus sylvatica* trees and maize crop as vegetative border structures, respectively. Likewise, Lazzaro et al. (2008) emphasise the role of optical porosity for the interception rate in hedgerows regarding spray drift.

In general, mustard crops form markedly denser vegetation layers in terms of plants per area compared to rape crops. However, the present data do not allow a definite conclusion on the mechanisms behind the elevated mass-related residues in mustard flowers compared to rape flowers, i.e. whether they are a result of different vegetation densities, scavenger characteristics of the flowers, or both.

### Impact of wind conditions on a.s. deposition

Wind speed and wind direction during drilling was subject to variation to some extent. Generally, for small and intermediate dust size fractions, higher wind velocities lead to more spread-out dust deposition patterns, and longer peak deposition distances from the dust emission source (Devarrewaere et al. 2016b). This is reflected by the results presented here. However, efforts have been made on keeping wind characteristics as constant as possible. Moreover, although Krupke et al. (2017) found a significant effect of wind direction on dust deposition, they observed a low amount of variance explained by this factor, at least for relatively low wind speed ( < 13 km/h) which are in the same range as in our experiments.

## Conclusion

The present work highlights several key factors which have to be taken into account in the exposure assessment of NTAs in fields adjacent to the drilling of treated seeds. The Heubach a.s. value (expressed as g a.s. per ha; HVAS) is considered as a global, i.e. scenario-independent, measure of potential dust deposition into adjacent fields. Although the present results indicate that seed drilling technique has an impact on a.s. deposition in adjacent areas, further studies would be necessary to corroborate the present findings.

Petri dish samplers, which have been established as a standard method for measuring a.s. deposition, can be regarded as representative of the results from the plant samplers for a given combination of drilling technique and adjacent crop type. The factors derived in the present study may offer an opportunity to extrapolate from results from Petri dish samplers to a.s. residues on plant material when direct assessment of the latter is unfeasible.

The presence and type of the adjacent crop have an effect on a.s. deposition. Therefore, crop type and crop biomass should be taken into account in the exposure assessment for epigeic or ground exposed NTAs as well as NTA organisms present in nonflowering plant parts, but especially for flower visiting NTAs such as bees.

## Supplementary Information

ESM 1 (PDF 1599 kb)

## Data Availability

The datasets generated and analysed during the current study are available in the OpenAgrar repository, 10.5073/20210318-133039.
