# Supplementary material for: Deposition of dust with active substances in pesticides from treated seeds in adjacent fields during drilling: disentangling the effects of various factors using an 8-year field experiment
Source: Environ Sci Pollut Res Int. 2021 Jul 7;28(47):66613–27. doi: 10.1007/s11356-021-15247-w (PMC8642378; doi:10.1007/s11356-021-15247-w)
Supplement: Supplementary file 1 — (PDF 1599 kb) [file 11356_2021_15247_MOESM1_ESM.pdf]

Supplement to:

Deposition of dust with active substances in pesticides from treated seeds in adjacent fields during drilling: disentangling the effects of various factors using a 8-year field experiment

Environmental Science and Pollution Research

André Krahner[1*], Udo Heimbach[2], Matthias Stähler[3], Gabriela Bischoff[1], Jens Pistorius[1]

[1]Julius Kühn Institute (JKI) – Federal Research Centre for Cultivated Plants, Institut for Bee Protection, Braunschweig, Germany
[2]Julius Kühn Institute (JKI) – Federal Research Centre for Cultivated Plants, Institute for Plant Protection in Field Crops and Grassland, Braunschweig, Germany
[3]Julius Kühn Institute (JKI) – Federal Research Centre for Cultivated Plants, Institute for Ecological Chemistry, Plant Analysis and Stored Product Protection, Berlin, Germany
*Corresponding author: André Krahner, andre.krahner@julius-kuehn.de

S1 Chemical residue analysis

Summary and introduction

In the investigations, the active substance (a.s.) content of the synthetically produced substance class of neonicotinoids was analysed on passive collectors, plant parts (leaves and flowers) and Heubach filters. Three analytes were examined separately, each of which was assigned two internal isotope-labelled standards (IS). Table S1 gives an overview of all dust drift studies discussed in this article.

Table S1. Overview about treatments (neonicotinoid active substance (a.s.) and corresponding plant protection product (PPP)), off-crop, and internal standards (IS).

Code JKI	Sowing	PPP	Adjacent Off-Crop	a.s.	Surrogate Standard (IS A)	IS B
1008	maize	Poncho®	oilseed rape	clothianidin	acetamiprid-d3	imidacloprid-d4
1115	maize	Poncho®	oilseed rape	clothianidin	acetamiprid-d3	clothianidin-d3
1205	maize	Poncho Pro®	oilseed rape	clothianidin	acetamiprid-d3	clothianidin-d3
1122	oilseed rape	Elado®	mustard	clothianidin	acetamiprid-d3	clothianidin-d3
1315	oilseed rape	Elado®	mustard	clothianidin	acetamiprid-d3	clothianidin-d3
1411	oilseed rape	Elado®	mustard	clothianidin	acetamiprid-d3	clothianidin-d3
1405	spring oilseed rape	Elado®	oilseed rape	clothianidin	acetamiprid-d3	clothianidin-d3
1505	spring oilseed rape	Elado®	oilseed rape	clothianidin	acetamiprid-d3	clothianidin-d3
1224	barley	Gaucho® FS 600	mustard	imidacloprid	acetamiprid-d3	imidacloprid-d4
1636	spring wheat	Gaucho® FS 350	oilseed rape	imidacloprid	acetamiprid-d3	imidacloprid-d4
1647	winter wheat	Gaucho® FS 350	mustard	imidacloprid	acetamiprid-d3	imidacloprid-d4
1708	spring wheat	Gaucho® FS 350	oilseed rape	imidacloprid	acetamiprid-d3	imidacloprid-d4
1303	maize	Cruiser®	oilseed rape	thiamethoxam	clothianidin-d3	thiamethoxam-d4

Analytical methods to determine the active substances (analytes) clothianidin, thiamethoxam, and imidacloprid

Before the actual dust drift test in the field, the Heubach filters, stored in glass tubes, were sent to the laboratory to determine the a.s. content in the dust drift.

Shortly before the start of the experiment, untreated plant samples were taken for the recovery experiments. After sowing, plant parts (in aluminium foil or in 500 ml aluminium boxes) as well as the

Petri dishes/filter collectors (PFC) were sampled according to the experimental plan (Fig. S1). All samples arrived at the laboratory three hours after the end of the experiment and were frozen separately at - 18° C until they were processed.

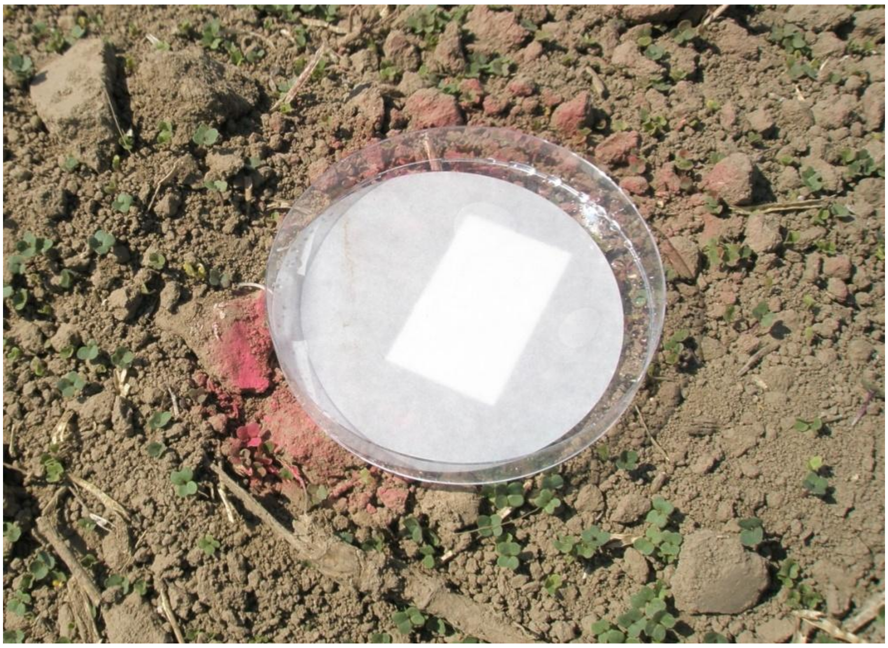

Fig. S1. Petri dish/filter collector (PFC) placed at the sampling point marker. Each PFC consisted of the lower part of a Petri dish (d=135 mm*20 mm, non-sterile), into which a filter paper (d=125 mm, type 602 H) was placed. The filter was moistened with a glycerine/water mixture 1/1 (v/v) for better fixation of the dust particles (glycerine 86% for analysis). After sampling and for transport to the analytical laboratory, the collector was covered with the lid of the Petri dish (d=145 mm*9 mm, non-sterile). The label on the backside of the PFC was labelled with the trial code and the sample number. All PFC were prepared in the laboratory. Photo: © Institute for Ecological Chemistry, Plant Analysis and Stored Product Protection, Julius Kühn Institute.

The analytical methods ~~a~~re described below.

Heubach a.s. method – determination of a.s. on filters

- Add the surrogate acetamiprid-d3 (IS A) to each filter sample in the sampling tube.
- Then extract with 10 ml acetone for 10 min in an ultrasonic bath, followed by 50 min on a circular shaker at 560 rpm.
- Isotopically labelled internal standards were added depending on the analyte (imidacloprid-d4, clothianidin-d3 or thiamethoxam-d4). The internal standard solution (IS B) was used to adjust the extracts to a final volume as well as to dilute them 1:10.
- The analyte content was determined by LC-MS/MS technique.

Extraction of Petri-dish/filter collector (PFC), (filter wetted with glycerol/water mixtures 1:1 (v/v))

- Filters were folded and placed into 100 ml Duran glass bottles. Adding a surrogate standard solution (internal standard (IS A)) to sample.
- The Petri dishes were rinsed with about 60 ml of methanol to quantitatively transfer the sample into the same Duran glass bottle.
- Each sample was extracted for 10 minutes using an ultrasonic bath and then transferred quantitatively with 30 ml acetone into 250 ml flat bottom flasks.
- Extracts were concentrated to the aqueous residue using a rotary evaporator.
- This aqueous extract was re-dissolved with pure water, transferred in a glass tube.
- Flat bottom flasks were rinsed with 2 ml dichloromethane, the solvent transferred in the same tube and shaken out (total three times) to eliminate glycerol.
- All extracts were combined in another tube and evaporated to dryness using nitrogen.

- The residual extract was re-dissolved with 1.0 ml methanol/water-mixture containing the internal standard (IS B).
- The filtered (0.20 µm) extract was analysed by LC-MS/MS.

Extraction oil seed rape or mustard leaves and open flowers of plants wetted with glycerol/water mixtures (1:1, v/v)

- Preparation of the plant sample (whole sample of 0.1 m^2 sample area) for homogenization.
- After adding a surrogate standard solution (IS A) and 150 ml acetone/water-mixture (3:1, v/v) the plant sample were homogenized for 3 minutes with a Warring Blender (mixer); then filtered, rinsed and transferred with 70 ml of the acetone/water-mixture (3:1, v/v) and made up to 250 ml.
- An aliquot of 50 ml was used for further analysis.
- Organic solvent was removed with a rotary evaporator and the aqueous residue (10 ml-15 ml) made up to 20 ml with pure water.
- Solid liquid extraction with 100 ml dichloromethane using ChemElut® cartridges (VARIAN 12198008).
- Evaporating the extract to dryness using a rotary evaporator and dissolving the residue with acetonitrile containing IS B; final volume 2 ml.
- This extract was filtered (0.20 µm) and analysed by LC-MS/MS. (If necessary the extract was diluted with internal standard solution).

LC-MS/MS Determination

The quantification and identification of the different substances on PFC and plants was carried out with an UltiMate 3000 HPLC system (Dionex, since 2011, Thermo Fisher Scientific, Sunnyvale, CA, USA) coupled to a QTRAP 5500 triple quadrupole mass spectrometer (SCIEX, Framingham, MA, USA), equipped with an electrospray ionisation (ESI) source. Tables S2 and S3 summarize essential device and working parameters of the LC-MS/MS measuring system.

Validation of the analysis methods

The method was validated by fortification experiments and the calculation of recovery rates and relative standard deviations (coefficients of variation) based on the measuring data for each fortification level. At each fortification level the mean recovery rates should be in the range of 60% to 120% with a relative standard deviation of ≤ 30% *(mean recovery and precision criteria for concentration level > 1 µg/kg ≤ 0.01 mg/kg, Guidance Document on Pesticide Residue Analytical Methods, SANCO/825/00 rev. 8.1, 16/11/2010)*.

The method validation for the determination of clothianidin on PFC and plants parts (open flowers and leaves) was performed via fortification experiments and the results are presented in Table S4. Example chromatograms of a calibration standard solution, PFC sample, and plant matrix sample are shown in Fig. S2-S5.

Analogously, the results of the recovery experiments for imidacloprid are compiled in Table S5 and for thiamethoxam in Table S6. Fig. S6 and S7 show typical chromatograms of an imidacloprid calibration standard solution and PFC sample.

Table S2. Parameters of the liquid chromatography (LC)

LC	UltiMate 3000 RS (Dionex)			
Column	Phenomenex Kinetex Phenyl-Hexyl 50 mm, i.d.: 2.1 mm, partical size: 2.6 µm			
Injection Volume	2 µl			
Column temperature	60 °C			
Solvent A	methanol + 0,1% formic acid			
Solvent B	water + 0,1% formic acid			
Gradient:	Time [min]	Flow rate [µl/min]	Solvent A [%]	Solvent B [%]
	0.0	500	2	98
	2.0	500	100	0
	3.0	500	100	0
	3.01	500	2	98
	4.0	500	2	98

Table S3. Parameter of mass spectrometry (MS)

Device	QTRAP 5500 (AB SCIEX)				
Software	Analyst 1.6.1				
Scan Type	MRM				
Polarity	Positive				
Curtain gas	40 psi				
Temperature	450 °C				
Auxiliary gas	50 psi				
Nebulizing gas	80 psi				
Spray voltage	+ 5500 V				
Ionization	Elektrospray Ionization (ESI)				
Monitoring-Ionen:	a.s.	Q 1 Mass [amu]	Q 3 / 1 (Quantifier) Mass [amu]	Dwell time [ms]	Q 3 / 2 (Qualifier) Mass [amu]
Analyte 1	clothianidin	250	169	100	132
Surrogate (IS A) 1	acetamiprid-d3	226	126	100	
IS B 1	clothianidin-d3	253	172	100	
Analyte 2	thiamethoxam	292	211	100	181
Surrogate (IS A) 2	clothianidin-d3	253	172	100	
IS B 2	thiamethoxam-d4	296	215	100	
Analyte 3	imidacloprid	256	70	50	175
Surrogate (IS A) 3	acetamiprid-d3	226	126	100	
IS B 3	imidacloprid-d4	260	213	50	
Quantification	relative peak areas				
Calibration [pg/µl]	0.10, 0.50, 1.0, 5.0, 10, 25, 50, 100				
MS Detection Limit [pg/µl]	0.10				

Table S4. Method validation for the determination of clothianidin on Petri-dish/filter collector (PFC) with an area of PFC=0.0143 m² and plant parts of oil seed rape or mustard

	Clothianidin				Acetamiprid-d3 (surrogate)			
Matrix	Fortification Level	N	Recovery [%]	RSD [%]	EC [ng/ml]	Recovery [%]	RSD [%]	Code JKI
PFC	0,001 µg/PFC	4	108	8.9	10	81	1.4	1008
	0,010 µg/PFC	4	59	4.8	10	82	4.3	1008
	0,020 µg/PFC	4	77	4.1	10	75	1.3	1008
	0,002 µg/PFC	4	66	1.9	50	113	2.4	1205
	0,005 µg/PFC	8	70	9.7	50	102	6.0	1205
	0,010 µg/PFC	8	59	7.7	50	104	4.7	1205
Open Flowers	0.10 µg/0.096 m²	4	80	11.0	100	95	2.9	1008
	0.50 µg/0.096 m²	2	94	-	100	96	-	1008
	0.50 µg/0.096 m²	2	101	-	50	86	-	1115
	2.5 µg/0.096 m²	3	98	2.1	50	93	3.7	1115
	0.10 µg/0.096 m²	3	81	2.8	20	80	8.8	1205
	1.0 µg/0.096 m²	3	96	3.1	20	80	8.8	1205
	5.0 µg/0.096 m²	3	91	2.9	20	96	1.2	1205
Leaves	0.10 µg/0,096 m²	4	88	11.3	100	88	4.6	1008
	0.50 µg/0,096 m²	4	59	21.0	100	76	11.0	1008
	0.50 µg/0.096 m²	3	83	7.9	200	107	2.4	1115
	1.0 µg/0.096 m²	3	104	9.5	200	114	3.9	1115
	0.10 µg/0.096 m²	2	82	-	50	74	-	1205
	1.0 µg/0.096 m²	3	89	5.3	50	73	4.4	1205
	5.0 µg/0.096 m²	3	87	2.9	50	75	2.3	1205

RSD = Relative Standard Deviation, N = Number of replicates, EC = Expected Concentration in Final Volume

Table S5. Method validation for the determination of imidacloprid and acetamiprid-d3 on PFC with an area of PFC=0.0143 m², mustard leaves and flowers with surrogate acetamiprid-d3.

		Imidacloprid			Aetamiprid-d3 (Surrogate)			
Matrix	Level	N	Recovery [%]	RSD [%]	N	Recovery [%]	RSD [%]	Code JKI
PFC	0.010 µg/PFC	3	87	3.3	3	115	2.6	1224
	0.050 µg/PFC	3	83	2.5	3	95	2.8	1224
Open Flowers	0.010 µg/0.096 m²	3	127	3.5	3	111	6.2	1224
	0.050 µg/0.096 m²	3	116	1.6	3	114	3.1	1224
	0.100 µg/0.096 m²	3	100	6.1	3	101	9.5	1224
Leaves	0.010 µg/0.096 m²	3	81	6.9	3	125	9.0	1224
	0.050 µg/0.096 m²	3	90	1.6	3	111	13.0	1224
	0.100 µg/0.096 m²	3	89	6.7	3	79	16	1224

RSD = Relative Standard Deviation, N = Number of replicates

Table S6. Method validation for the determination of thiamethoxam on Petri-dish/filter collector (PFC=0.0143 m²) and plant (oil seed rape).

	Thiamethoxam				Clothianidin-d3 (Surrogate)			
Matrix	Fortification Level	N	Recovery [%]	RSD [%]	N	Recovery [%]	RSD [%]	Code JKI
PFC	0.001 µg/PFC	3	108	5.1	3	50	6.3	1303
	0.010 µg/PFC	2	83	-	2	52	-	1303
	0.100 µg/PFC	3	79	0.66	3	52	1.3	1303
Leaves	0.010 µg/0.096 m²	3	114	4.2	3	98	2.6	1303
	0.050 µg/0.096 m²	3	92	3.0	3	96	2.2	1303
	0.100 µg/0.096 m²	3	93	1.6	3	99	0.24	1303
Open Flowers	non flowering plants, oilseed rape, BBCH 76							1303

RSD = Relative Standard Deviation, N = Number of replicates

The fortification experiments were carried out in the years 2010 to 2013 and adapted to the analysis objectives. In addition to the analyte results (recovery rates, RSD), the surrogate standard results were also reported in the tables. The individual data including the number of replicates for the three a.s. and the two surrogates used in or on the matrices can be taken from the tables.

The Heubach a.s. analysis can be compared to the PFC recovery test results, as both involve the extraction of a filter. The data of the surrogate standard (IS B) for each sample also validate the method. In addition, there was the successful participation in a ring test ESA (European Seed Association) "Determination of imidacloprid, clothianidin and thiamethoxam on Heubach filters" in 2014, which enables a laboratory comparison (internal code "JKI 1322").

The concentration range for the recovery studies was chosen to cover the range of field data.

Regarding a.s. clothianidin, the analytical possibilities for the plant parts were not exhausted. Considering a measured value of 1.0 pg/µl from the calibration curve, a content of 0.010 µg clothianidin/0.096m² on leaves or flowers could be calculated. This was validated only for imidacloprid and thiamethoxam in the recovery experiments.

On the other hand, for imidacloprid the analytical possibilities for the PFCs were not exhausted. Considering a measured value of 1.0 pg/µl from the calibration curve, a content of 0.0010 µg/PFC could be calculated for these analytes. This was validated for clothianidin and thiamethoxam in the recovery experiments.

Calculation of the analysis results

For quantification, the internal standard method was used with an appropriate isotope-labelled internal standard for the target analyte using a calibration curve of 0.10 pg/µl to 100 pg/µl (Fig. S8-S10) and matrix-matched calibration standards. This method minimises the matrix effect and allows accurate quantification. In the case of sample dilution, sample extracts were quantified with isotope-labelled standards in solvent. Deuterated surrogate standards were added to each sample for internal monitoring of analytical quality.

The methods were validated via recovery studies by analysing a series of spiked replicate samples from Petri dishes and plant parts and determining the recovery rates of the target analytes. Limits of detection (LOD) and limits of quantification (LOQ) were determined experimentally from the recovery studies where possible and additionally estimated from the signal-to-noise ratios of the matrix standards.

Two multiple reaction monitoring (MRM) transitions were used to confirm the identity of each analyte and to determine the respective LOD and LOQ. This approach was sufficient, because the target compounds were known and only one was analysed at a time.

The LOQ values of all target substances from the neonicotinoid group were determined at 0.070 µg/m² for the PFC and at 0.10 µg/m² for the plant parts open flowers or leaves. The corresponding LODs of all a.s. and matrices were calculated from the lowest concentration level of the calibration curves and were one tenth below the LOQ.

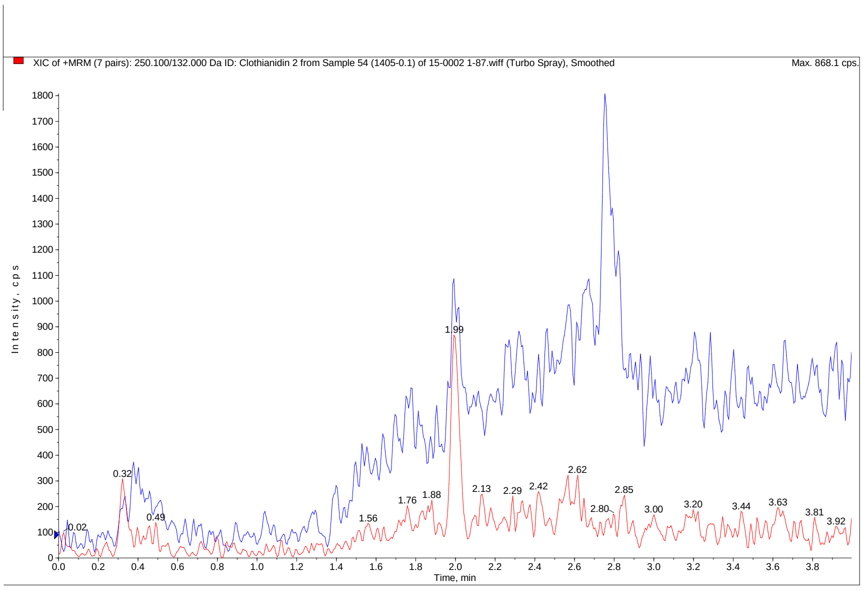

Fig. S2. An LC-MS/MS chromatogram of calibration standard solution with 0.10 ng/ml clothianidin (LOD, RT=1.99 min, blue line = quantifier ion, red line = qualifier ion) (s. Table S3). The analysis values were calculated by the internal standard method using a calibration curve of 0.10 pg/µl to 100 pg/µl.

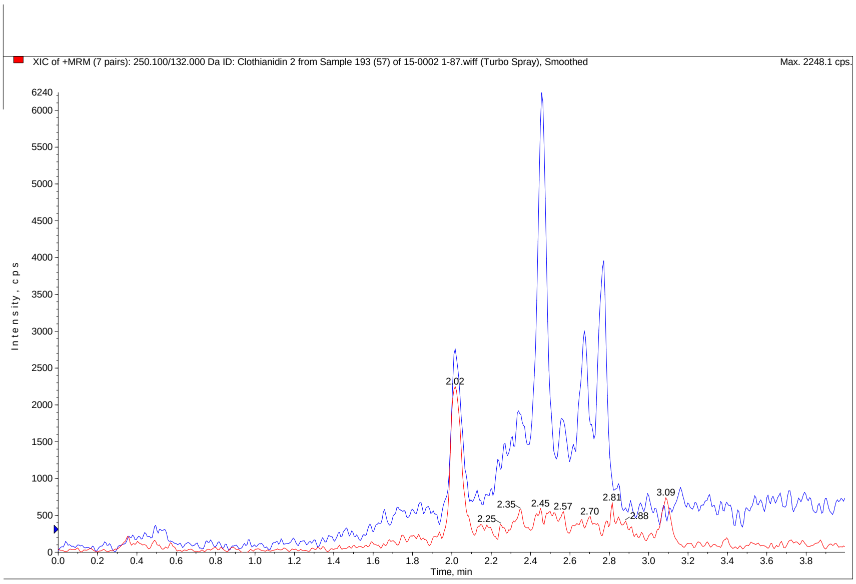

Fig. S3. Typical LC-MS/MS chromatogram of a PFC sample with 0.79 ng/ml clothianidin. (RT=2.02 min, blue line = quantifier ion, red line = qualifier ion).

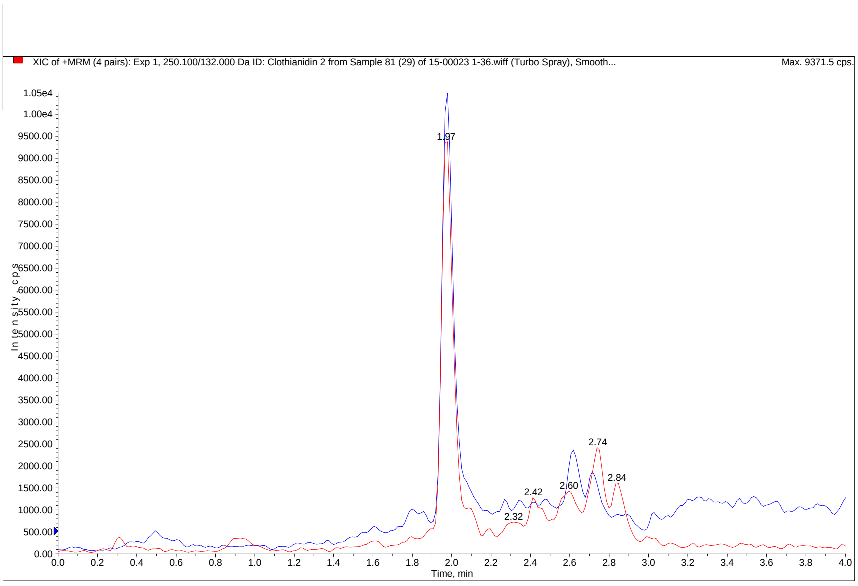

Fig. S4. LC-MS/MS chromatogram of a flower sample of oilseed rape with 2.5 ng/ml clothianidin (RT=1.97 min, blue line = quantifier ion, red line = qualifier ion).

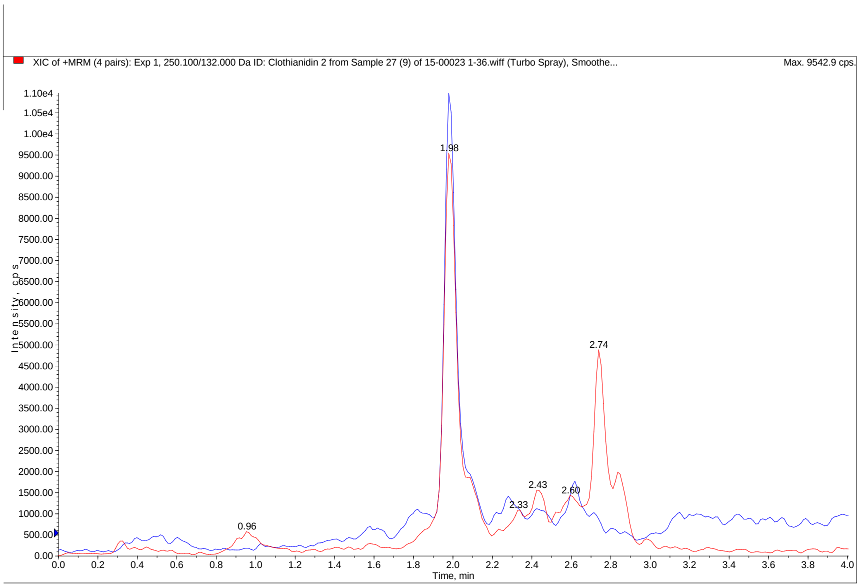

Fig. S5. LC-MS/MS chromatogram of a leave sample of oilseed rape with 0.31 ng/ml clothianidin, RT=1.98 min, (RT=2.02 min, blue line = quantifier ion, red line = qualifier ion).

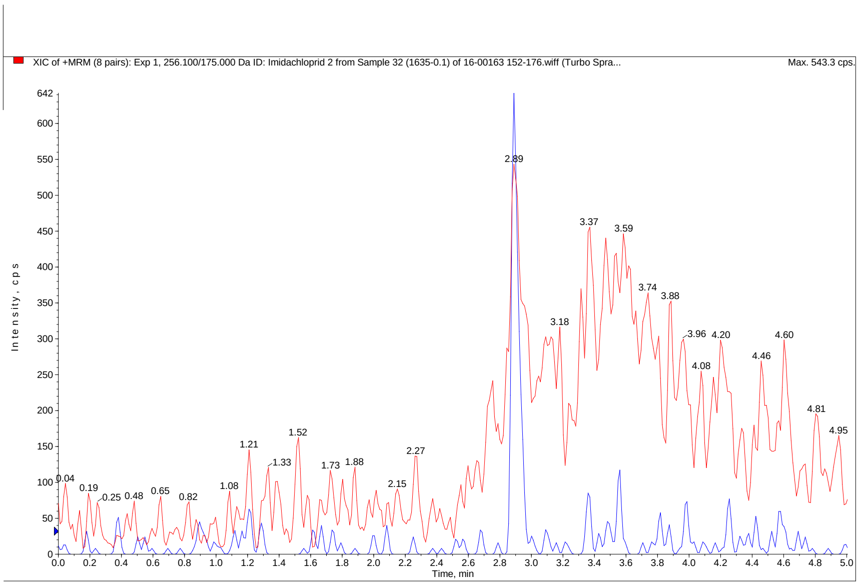

Fig. S6. Chromatogram of a calibrated standard solution of imidacloprid 0.10 ng/ml. (LOD, RT=2.89 min, blue line = quantifier ion, red line = qualifier ion).

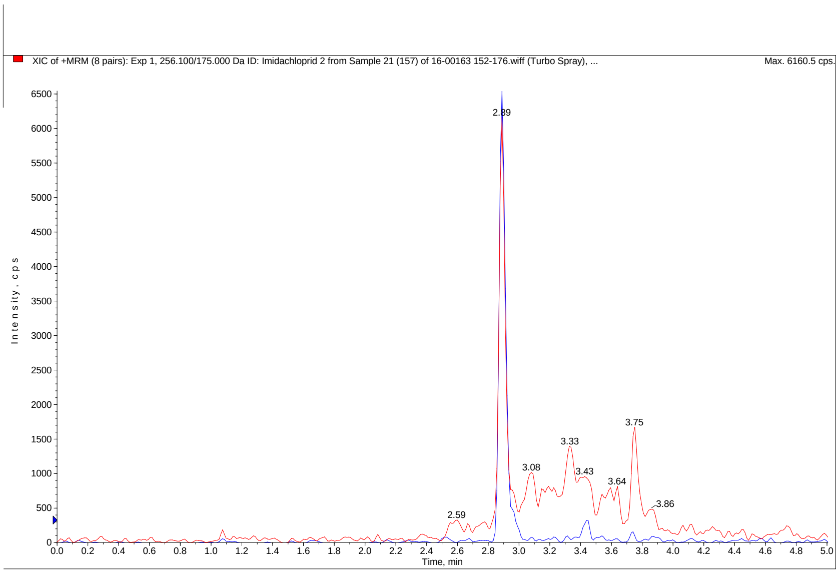

Fig. S7. An example chromatogram of a PFC sample with 2.6 ng/ml imidacloprid. (RT=2.89 min, blue line = quantifier ion, red line = qualifier ion).

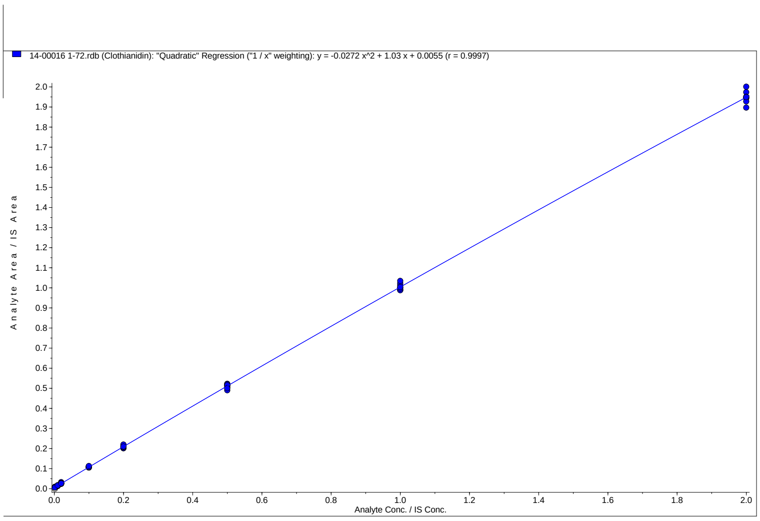

Fig. S8. Typical calibration curve of clothianidin with y=-0.0272x²+1.03x+0.0055 (r=0.9997). The analysis values were calculated by the internal standard method using a calibration curve of 0.1 pg/µl to 100 pg/µl.

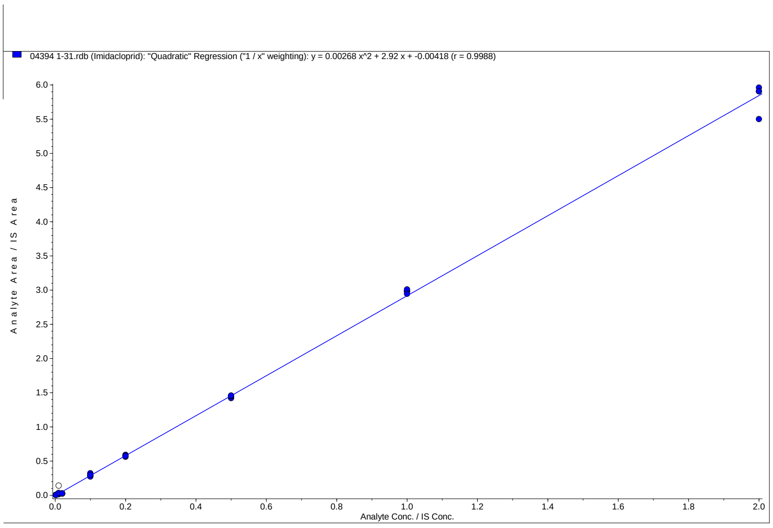

Fig. S9. Typical calibration curve of imidacloprid with y=0.00268x²+2.92x-0.00418 with r=0.9988. The analysis values were calculated by the internal standard method using a calibration curve of 0.1 pg/µl to 100 pg/µl.

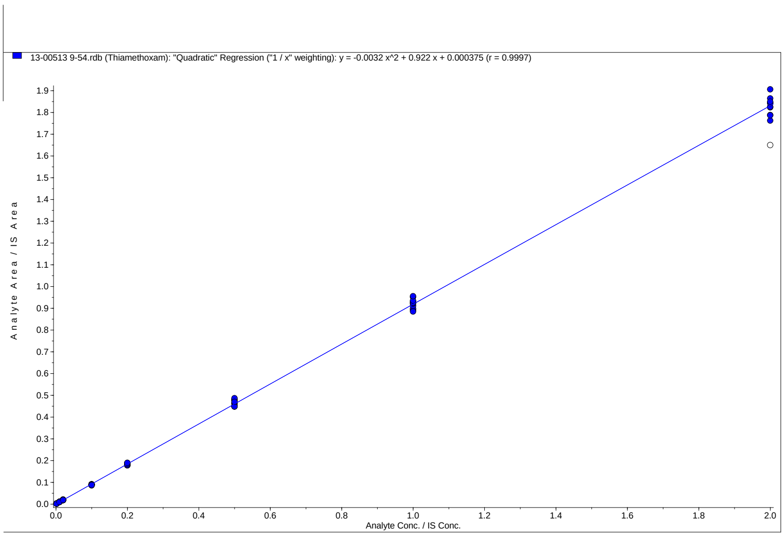

Fig. S10. Typical calibration standards curve from thiamethoxam with y=-0.00x²+0.922+0.000375 and r=0.9997. The analysis values were calculated by the internal standard method using a calibration curve of 0.1 pg/µl to 100 pg/µl.

S2 Wind conditions during drilling

Wind speed and direction during the drilling procedure is shown in the wind rose plots in Fig. S11.

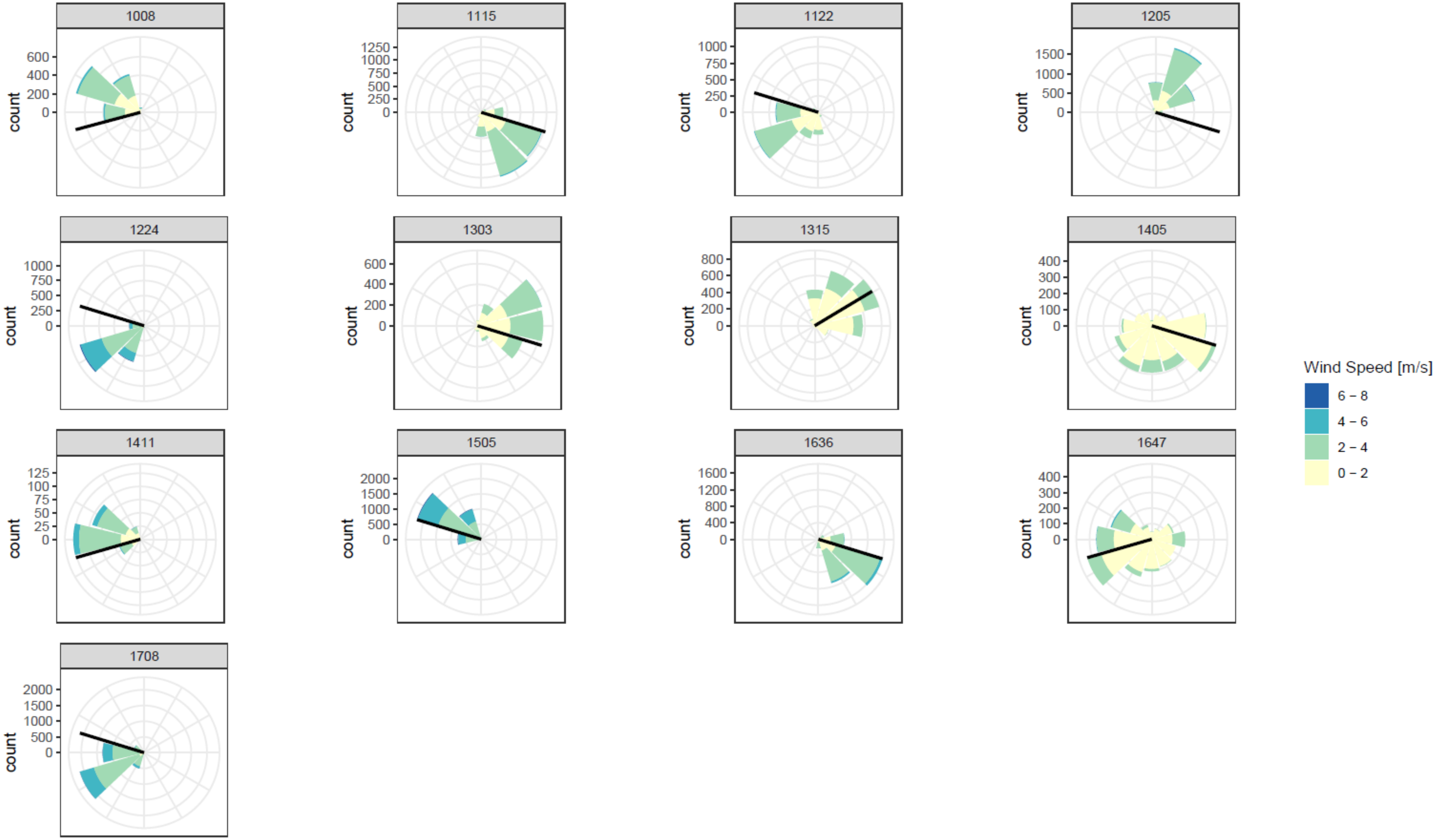

Fig. S11. Wind rose plots showing wind speed and wind direction for single trials (trial codes shown) during the drilling procedure, relative to the experimental target direction (black line). For trial codes refer to Table 1. Note that intervals of wind measurements varied between the trials, resulting in differences in total counts.

S3 Residue measurements in upwind direction

Residues measured in samples collected from upwind direction with regard to the drilled field (3 m from the field edge) are shown in Fig. S12.

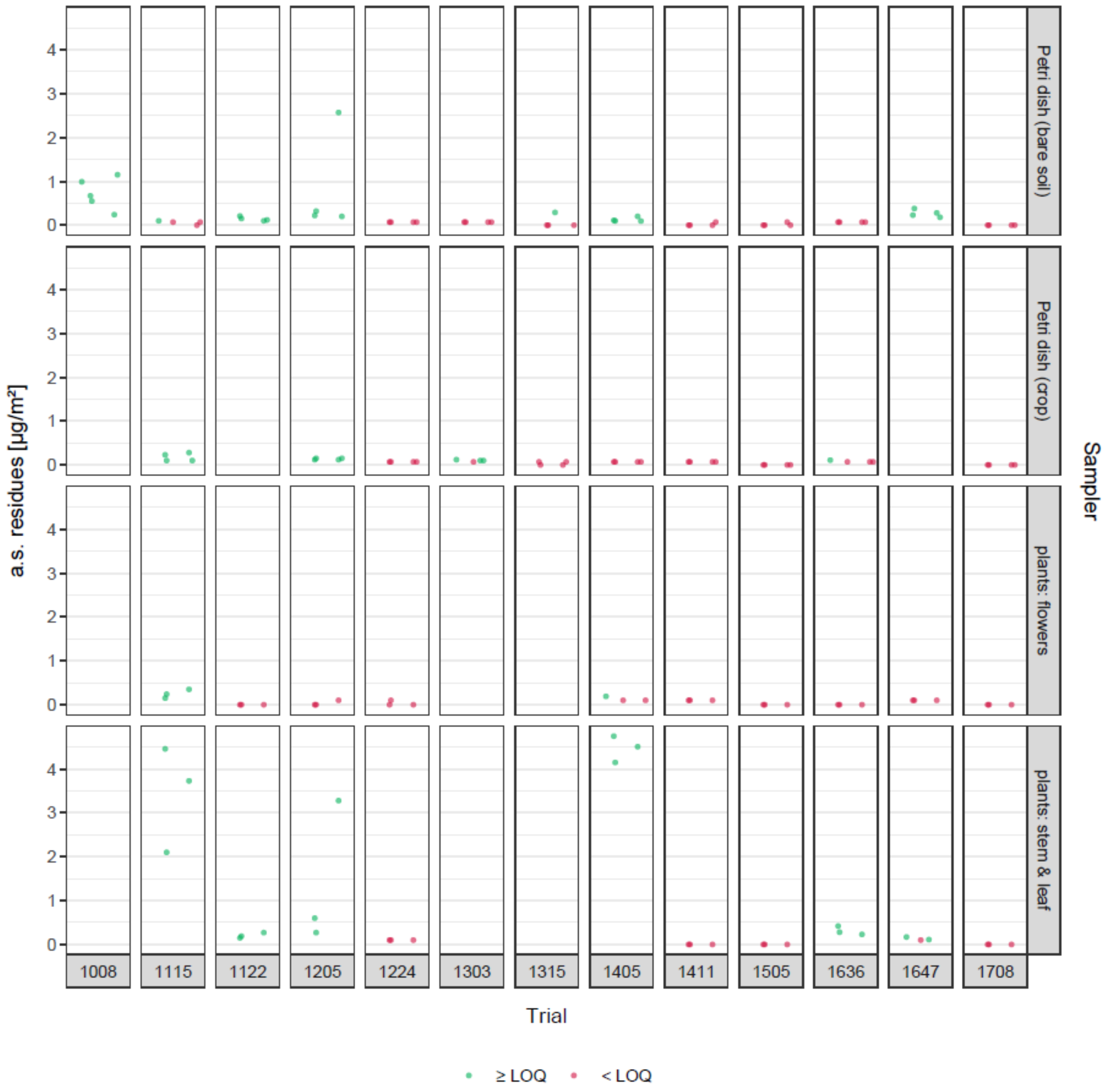

Fig. S12. Residues from samples collected from plants (flowers; stems and leaves) and petri dishes with filter collectors in upwind direction with regard to the drilled field. Points are jittered along the x-axis for improved readability.

S4 Results from GLMMs and post-hoc pairwise comparisons

Table S7. GLMM results for the Petri-dish-only dataset (n = 1218 observations), showing fitted mean values (response; µg a.s./m²), standard error (SE), degrees of freedom (df), asymptotic lower and upper 95% confidence limits (asymp.LCL and asymp.UCL, respectively). Effects: distance from the edge of the drilling area, combination of drilling method and adjacent crop (DMAC; ap = air pressure, as = air suction).

distance [m]	DMAC	response	SE	df	asymp.LCL	asymp.UCL
0	ap_bare_soil	2.785	1.151	Inf	1.240	6.259
1	ap_bare_soil	2.241	0.926	Inf	0.998	5.035
3	ap_bare_soil	1.769	0.731	Inf	0.787	3.976
5	ap_bare_soil	1.32	0.545	Inf	0.588	2.965
0	ap_mustard	2.053	0.910	Inf	0.861	4.894
1	ap_mustard	0.867	0.384	Inf	0.364	2.066
3	ap_mustard	0.639	0.283	Inf	0.268	1.524
5	ap_mustard	0.546	0.242	Inf	0.229	1.300
0	ap_rape	1.669	0.713	Inf	0.722	3.857
1	ap_rape	0.825	0.353	Inf	0.357	1.908
3	ap_rape	0.379	0.162	Inf	0.164	0.876
5	ap_rape	0.292	0.125	Inf	0.126	0.675
0	as_bare_soil	19.09	9.227	Inf	7.405	49.229
1	as_bare_soil	9.524	4.546	Inf	3.737	24.273
3	as_bare_soil	7.717	3.693	Inf	3.021	19.714
5	as_bare_soil	5.622	2.690	Inf	2.201	14.361
0	as_rape	14.62	7.201	Inf	5.571	38.391
1	as_rape	4.348	2.141	Inf	1.657	11.413
3	as_rape	2.093	1.030	Inf	0.797	5.492
5	as_rape	1.394	0.686	Inf	0.531	3.656

Table S8. GLMM results for the multi-sampler dataset (n = 1390 observations), showing fitted mean values (response; µg a.s./m²), standard error (SE), degrees of freedom (df), asymptotic lower and upper 95% confidence limits (asymp.LCL and asymp.UCL, respectively). Effects: distance from the edge of the drilling area, sampler, combination of drilling method and adjacent crop (DMAC; ap = air pressure, as = air suction).

distance [m]	sampler	DMAC	response	SE	df	asymp.LCL	asymp.UCL
0	Petri dish	ap_mustard	1.524	0.685	Inf	0.632	3.676
1	Petri dish	ap_mustard	0.813	0.366	Inf	0.337	1.964
3	Petri dish	ap_mustard	0.445	0.200	Inf	0.185	1.072
5	Petri dish	ap_mustard	0.452	0.203	Inf	0.187	1.091
0	plants: flowers	ap_mustard	1.457	0.660	Inf	0.600	3.541
1	plants: flowers	ap_mustard	0.778	0.351	Inf	0.321	1.884
3	plants: flowers	ap_mustard	0.426	0.192	Inf	0.176	1.029
5	plants: flowers	ap_mustard	0.432	0.195	Inf	0.179	1.045
0	plants: stem & leaf	ap_mustard	2.859	1.287	Inf	1.183	6.908
1	plants: stem & leaf	ap_mustard	1.525	0.687	Inf	0.631	3.688
3	plants: stem & leaf	ap_mustard	0.835	0.377	Inf	0.345	2.025
5	plants: stem & leaf	ap_mustard	0.848	0.383	Inf	0.350	2.057
0	Petri dish	ap_rape	0.928	0.372	Inf	0.423	2.036
1	Petri dish	ap_rape	0.431	0.173	Inf	0.197	0.945
3	Petri dish	ap_rape	0.202	0.081	Inf	0.092	0.444
5	Petri dish	ap_rape	0.156	0.062	Inf	0.071	0.342
0	plants: flowers	ap_rape	1.199	0.482	Inf	0.546	2.635
1	plants: flowers	ap_rape	0.557	0.224	Inf	0.253	1.225
3	plants: flowers	ap_rape	0.262	0.105	Inf	0.119	0.575
5	plants: flowers	ap_rape	0.202	0.081	Inf	0.092	0.443
0	plants: stem & leaf	ap_rape	2.432	0.977	Inf	1.107	5.345
1	plants: stem & leaf	ap_rape	1.129	0.454	Inf	0.514	2.482
3	plants: stem & leaf	ap_rape	0.530	0.213	Inf	0.241	1.166
5	plants: stem & leaf	ap_rape	0.409	0.164	Inf	0.186	0.899
0	Petri dish	as_rape	18.376	8.292	Inf	7.588	44.499
1	Petri dish	as_rape	6.917	3.130	Inf	2.850	16.791
3	Petri dish	as_rape	3.385	1.534	Inf	1.393	8.229
5	Petri dish	as_rape	2.678	1.214	Inf	1.101	6.513
0	plants: flowers	as_rape	34.895	15.917	Inf	14.272	85.316
1	plants: flowers	as_rape	13.136	5.987	Inf	5.376	32.094
3	plants: flowers	as_rape	6.428	2.924	Inf	2.636	15.676
5	plants: flowers	as_rape	5.085	2.310	Inf	2.087	12.388
0	plants: stem & leaf	as_rape	35.387	16.113	Inf	14.496	86.384
1	plants: stem & leaf	as_rape	13.321	6.046	Inf	5.472	32.426
3	plants: stem & leaf	as_rape	6.519	2.966	Inf	2.672	15.903
5	plants: stem & leaf	as_rape	5.157	2.344	Inf	2.116	12.568

Table S9. GLMM results for the plant-material only dataset (n = 833 observations), showing fitted mean values (response; a.s./kg), standard error (SE), degrees of freedom (df), asymptotic lower and upper 95% confidence limits (asymp.LCL and asymp.UCL, respectively). Effects: distance from the edge of the drilling area, sampler, combination of drilling method and adjacent crop (DMAC; ap = air pressure, as = air suction).

distance [m]	sampler	DMAC	response	SE	df	asymp.LCL	asymp.UCL
0	flowers	ap_mustard	18.114	8.093	Inf	7.546	43.483
1	flowers	ap_mustard	8.281	3.693	Inf	3.455	19.846
3	flowers	ap_mustard	4.245	1.892	Inf	1.773	10.168
5	flowers	ap_mustard	4.177	1.859	Inf	1.746	9.994
0	stem & leaf	ap_mustard	2.251	1.002	Inf	0.940	5.388
1	stem & leaf	ap_mustard	1.058	0.471	Inf	0.442	2.534
3	stem & leaf	ap_mustard	0.441	0.197	Inf	0.184	1.060
5	stem & leaf	ap_mustard	0.387	0.173	Inf	0.161	0.927
0	flowers	ap_rape	3.675	1.460	Inf	1.686	8.007
1	flowers	ap_rape	1.680	0.669	Inf	0.770	3.665
3	flowers	ap_rape	0.861	0.343	Inf	0.395	1.880
5	flowers	ap_rape	0.847	0.339	Inf	0.387	1.854
0	stem & leaf	ap_rape	1.149	0.458	Inf	0.526	2.510
1	stem & leaf	ap_rape	0.540	0.215	Inf	0.248	1.179
3	stem & leaf	ap_rape	0.225	0.090	Inf	0.103	0.492
5	stem & leaf	ap_rape	0.197	0.079	Inf	0.090	0.431
0	flowers	as_rape	55.407	28.237	Inf	20.406	150.441
1	flowers	as_rape	25.328	12.924	Inf	9.317	68.854
3	flowers	as_rape	12.986	6.623	Inf	4.779	35.284
5	flowers	as_rape	12.775	6.511	Inf	4.705	34.690
0	stem & leaf	as_rape	14.508	7.396	Inf	5.342	39.403
1	stem & leaf	as_rape	6.822	3.480	Inf	2.510	18.540
3	stem & leaf	as_rape	2.843	1.449	Inf	1.047	7.722
5	stem & leaf	as_rape	2.492	1.271	Inf	0.917	6.774

Table S10. Post-hoc test results for the Petri-dish-only dataset (n = 1218 observations) showing contrasts between distances from the edge of the drilling area (SE = standard error, df = degrees of freedom). P values were adjusted using the Tukey method. DMAC: combination of drilling method and adjacent crop.

contrast	DMAC	ratio	SE	df	z.ratio	p.value
0 m / 1 m	air pressure & bare soil	1.243	0.101	Inf	2.672	3.780E-02
0 m / 3 m	air pressure & bare soil	1.574	0.129	Inf	5.519	2.040E-07
0 m / 5 m	air pressure & bare soil	2.110	0.173	Inf	9.122	4.940E-14
1 m / 3 m	air pressure & bare soil	1.267	0.104	Inf	2.887	2.033E-02
1 m / 5 m	air pressure & bare soil	1.698	0.139	Inf	6.478	5.584E-10
3 m / 5 m	air pressure & bare soil	1.340	0.110	Inf	3.574	1.997E-03
0 m / 1 m	air pressure & mustard	2.367	0.302	Inf	6.747	9.078E-11
0 m / 3 m	air pressure & mustard	3.211	0.414	Inf	9.049	5.318E-14
0 m / 5 m	air pressure & mustard	3.761	0.479	Inf	10.408	3.353E-14
1 m / 3 m	air pressure & mustard	1.356	0.172	Inf	2.406	7.587E-02
1 m / 5 m	air pressure & mustard	1.589	0.201	Inf	3.657	1.454E-03
3 m / 5 m	air pressure & mustard	1.171	0.149	Inf	1.244	5.985E-01
0 m / 1 m	air pressure & rape	2.023	0.229	Inf	6.224	2.899E-09
0 m / 3 m	air pressure & rape	4.400	0.486	Inf	13.413	0.000E+00
0 m / 5 m	air pressure & rape	5.718	0.638	Inf	15.628	0.000E+00
1 m / 3 m	air pressure & rape	2.175	0.244	Inf	6.913	2.855E-11
1 m / 5 m	air pressure & rape	2.826	0.326	Inf	9.004	2.676E-14
3 m / 5 m	air pressure & rape	1.300	0.146	Inf	2.333	9.062E-02
0 m / 1 m	air suction & bare soil	2.005	0.256	Inf	5.451	3.004E-07
0 m / 3 m	air suction & bare soil	2.474	0.326	Inf	6.880	3.589E-11
0 m / 5 m	air suction & bare soil	3.396	0.446	Inf	9.311	4.075E-14
1 m / 3 m	air suction & bare soil	1.234	0.124	Inf	2.100	1.529E-01
1 m / 5 m	air suction & bare soil	1.694	0.169	Inf	5.272	8.058E-07
3 m / 5 m	air suction & bare soil	1.373	0.146	Inf	2.979	1.532E-02
0 m / 1 m	air suction & rape	3.363	0.477	Inf	8.549	3.619E-14
0 m / 3 m	air suction & rape	6.988	0.993	Inf	13.684	0.000E+00
0 m / 5 m	air suction & rape	10.494	1.482	Inf	16.649	0.000E+00
1 m / 3 m	air suction & rape	2.078	0.293	Inf	5.184	1.297E-06
1 m / 5 m	air suction & rape	3.120	0.438	Inf	8.107	5.229E-14
3 m / 5 m	air suction & rape	1.502	0.210	Inf	2.905	1.922E-02

Table S11. Post-hoc test results for the Petri-dish-only dataset (n = 1218 observations) showing contrasts between combinations of drilling method and adjacent crop (DMAC; SE = standard error, df = degrees of freedom). P values were adjusted using the Tukey method. Distance: distance to the edge of the drilling area.

contrast	distance [m]	ratio	SE	df	z.ratio	p.value
air pressure & bare soil / air pressure & mustard	0	1.357	0.280	Inf	1.477	5.772E-01
air pressure & bare soil / air pressure & rape	0	1.669	0.302	Inf	2.825	3.805E-02
air pressure & bare soil / air suction & bare soil	0	0.146	0.099	Inf	-2.840	3.642E-02
air pressure & bare soil / air suction & rape	0	0.190	0.130	Inf	-2.425	1.087E-01
air pressure & mustard / air pressure & rape	0	1.230	0.325	Inf	0.783	9.355E-01
air pressure & mustard / air suction & bare soil	0	0.108	0.075	Inf	-3.198	1.207E-02
air pressure & mustard / air suction & rape	0	0.140	0.099	Inf	-2.792	4.182E-02
air pressure & rape / air suction & bare soil	0	0.087	0.060	Inf	-3.552	3.515E-03
air pressure & rape / air suction & rape	0	0.114	0.079	Inf	-3.135	1.482E-02
air suction & bare soil / air suction & rape	0	1.306	0.316	Inf	1.102	8.053E-01
air pressure & bare soil / air pressure & mustard	1	2.585	0.532	Inf	4.612	3.928E-05
air pressure & bare soil / air pressure & rape	1	2.717	0.495	Inf	5.483	4.175E-07
air pressure & bare soil / air suction & bare soil	1	0.235	0.158	Inf	-2.148	1.998E-01
air pressure & bare soil / air suction & rape	1	0.515	0.352	Inf	-0.969	8.690E-01
air pressure & mustard / air pressure & rape	1	1.051	0.278	Inf	0.188	9.997E-01
air pressure & mustard / air suction & bare soil	1	0.091	0.063	Inf	-3.458	4.945E-03
air pressure & mustard / air suction & rape	1	0.199	0.140	Inf	-2.294	1.468E-01
air pressure & rape / air suction & bare soil	1	0.087	0.059	Inf	-3.586	3.096E-03
air pressure & rape / air suction & rape	1	0.190	0.131	Inf	-2.401	1.150E-01
air suction & bare soil / air suction & rape	1	2.190	0.511	Inf	3.363	6.900E-03
air pressure & bare soil / air pressure & mustard	3	2.768	0.573	Inf	4.921	8.528E-06
air pressure & bare soil / air pressure & rape	3	4.664	0.844	Inf	8.511	4.696E-14
air pressure & bare soil / air suction & bare soil	3	0.229	0.155	Inf	-2.184	1.857E-01
air pressure & bare soil / air suction & rape	3	0.845	0.578	Inf	-0.245	9.992E-01
air pressure & mustard / air pressure & rape	3	1.685	0.445	Inf	1.977	2.772E-01

Table S11 (continued).

contrast	distance [m]	ratio	SE	df	z.ratio	p.value
air pressure & mustard / air suction & bare soil	3	0.083	0.057	Inf	-3.589	3.062E-03
air pressure & rape / air suction & bare soil	3	0.049	0.034	Inf	-4.413	9.953E-05
air pressure & rape / air suction & rape	3	0.181	0.125	Inf	-2.468	9.800E-02
air suction & bare soil / air suction & rape	3	3.688	0.867	Inf	5.548	2.875E-07
air pressure & bare soil / air pressure & mustard	5	2.419	0.498	Inf	4.292	1.722E-04
air pressure & bare soil / air pressure & rape	5	4.521	0.823	Inf	8.286	5.573E-14
air pressure & bare soil / air suction & bare soil	5	0.235	0.158	Inf	-2.149	1.994E-01
air pressure & bare soil / air suction & rape	5	0.947	0.647	Inf	-0.079	1.000E+00
air pressure & mustard / air pressure & rape	5	1.869	0.493	Inf	2.370	1.235E-01
air pressure & mustard / air suction & bare soil	5	0.097	0.067	Inf	-3.361	6.949E-03
air pressure & mustard / air suction & rape	5	0.392	0.275	Inf	-1.334	6.699E-01
air pressure & rape / air suction & bare soil	5	0.052	0.035	Inf	-4.332	1.437E-04
air pressure & rape / air suction & rape	5	0.209	0.145	Inf	-2.259	1.583E-01
air suction & bare soil / air suction & rape	5	4.034	0.947	Inf	5.944	2.770E-08

Table S12. Post-hoc test results for the multi-sampler dataset (n = 1390 observations) showing contrasts between distances from the edge of the drilling area (SE = standard error, df = degrees of freedom). P values were adjusted using the Tukey method. DMAC: combination of drilling method and adjacent crop.

contrast	Sampler	DMAC	ratio	SE	df	z.ratio	p.value
0 m / 1 m	Petri dish	air pressure & mustard	1.874	0.228	Inf	5.157	1.500E-06
0 m / 3 m	Petri dish	air pressure & mustard	3.423	0.415	Inf	10.152	3.819E-14
0 m / 5 m	Petri dish	air pressure & mustard	3.372	0.414	Inf	9.896	3.686E-14
1 m / 3 m	Petri dish	air pressure & mustard	1.826	0.221	Inf	4.978	3.832E-06
1 m / 5 m	Petri dish	air pressure & mustard	1.799	0.221	Inf	4.780	1.042E-05
3 m / 5 m	Petri dish	air pressure & mustard	0.985	0.118	Inf	-0.126	9.993E-01
0 m / 1 m	plants: flowers	air pressure & mustard	1.874	0.228	Inf	5.157	1.500E-06
0 m / 3 m	plants: flowers	air pressure & mustard	3.423	0.415	Inf	10.152	3.819E-14
0 m / 5 m	plants: flowers	air pressure & mustard	3.372	0.414	Inf	9.896	3.686E-14
1 m / 3 m	plants: flowers	air pressure & mustard	1.826	0.221	Inf	4.978	3.832E-06
1 m / 5 m	plants: flowers	air pressure & mustard	1.799	0.221	Inf	4.780	1.042E-05
3 m / 5 m	plants: flowers	air pressure & mustard	0.985	0.118	Inf	-0.126	9.993E-01
0 m / 1 m	plants: stem & leaf	air pressure & mustard	1.874	0.228	Inf	5.157	1.500E-06
0 m / 3 m	plants: stem & leaf	air pressure & mustard	3.423	0.415	Inf	10.152	3.819E-14
0 m / 5 m	plants: stem & leaf	air pressure & mustard	3.372	0.414	Inf	9.896	3.686E-14
1 m / 3 m	plants: stem & leaf	air pressure & mustard	1.826	0.221	Inf	4.978	3.832E-06
1 m / 5 m	plants: stem & leaf	air pressure & mustard	1.799	0.221	Inf	4.780	1.042E-05
3 m / 5 m	plants: stem & leaf	air pressure & mustard	0.985	0.118	Inf	-0.126	9.993E-01
0 m / 1 m	Petri dish	air pressure & rape	2.154	0.229	Inf	7.202	3.577E-12
0 m / 3 m	Petri dish	air pressure & rape	4.586	0.482	Inf	14.484	0.000E+00
0 m / 5 m	Petri dish	air pressure & rape	5.952	0.633	Inf	16.783	0.000E+00
1 m / 3 m	Petri dish	air pressure & rape	2.129	0.227	Inf	7.081	8.588E-12
1 m / 5 m	Petri dish	air pressure & rape	2.763	0.297	Inf	9.461	3.475E-14
3 m / 5 m	Petri dish	air pressure & rape	1.298	0.138	Inf	2.459	6.657E-02
0 m / 1 m	plants: flowers	air pressure & rape	2.154	0.229	Inf	7.202	3.577E-12
0 m / 3 m	plants: flowers	air pressure & rape	4.586	0.482	Inf	14.484	0.000E+00
0 m / 5 m	plants: flowers	air pressure & rape	5.952	0.633	Inf	16.783	0.000E+00
1 m / 3 m	plants: flowers	air pressure & rape	2.129	0.227	Inf	7.081	8.588E-12
1 m / 5 m	plants: flowers	air pressure & rape	2.763	0.297	Inf	9.461	3.475E-14
3 m / 5 m	plants: flowers	air pressure & rape	1.298	0.138	Inf	2.459	6.657E-02
0 m / 1 m	plants: stem & leaf	air pressure & rape	2.154	0.229	Inf	7.202	3.577E-12
0 m / 3 m	plants: stem & leaf	air pressure & rape	4.586	0.482	Inf	14.484	0.000E+00
0 m / 5 m	plants: stem & leaf	air pressure & rape	5.952	0.633	Inf	16.783	0.000E+00
1 m / 3 m	plants: stem & leaf	air pressure & rape	2.129	0.227	Inf	7.081	8.588E-12
1 m / 5 m	plants: stem & leaf	air pressure & rape	2.763	0.297	Inf	9.461	3.475E-14
3 m / 5 m	plants: stem & leaf	air pressure & rape	1.298	0.138	Inf	2.459	6.657E-02
0 m / 1 m	Petri dish	air suction & rape	2.656	0.360	Inf	7.213	3.304E-12
0 m / 3 m	Petri dish	air suction & rape	5.428	0.741	Inf	12.399	0.000E+00
0 m / 5 m	Petri dish	air suction & rape	6.862	0.934	Inf	14.155	0.000E+00
1 m / 3 m	Petri dish	air suction & rape	2.043	0.277	Inf	5.274	7.960E-07
1 m / 5 m	Petri dish	air suction & rape	2.583	0.348	Inf	7.043	1.133E-11
3 m / 5 m	Petri dish	air suction & rape	1.264	0.170	Inf	1.739	3.032E-01
0 m / 1 m	plants: flowers	air suction & rape	2.656	0.360	Inf	7.213	3.304E-12

Table S12 (continued).

contrast	Sampler	DMAC	ratio	SE	df	z.ratio	p.value
0 m / 3 m	plants: flowers	air suction & rape	5.428	0.741	Inf	12.399	0.000E+00
0 m / 5 m	plants: flowers	air suction & rape	6.862	0.934	Inf	14.155	0.000E+00
1 m / 3 m	plants: flowers	air suction & rape	2.043	0.277	Inf	5.274	7.960E-07
1 m / 5 m	plants: flowers	air suction & rape	2.583	0.348	Inf	7.043	1.133E-11
3 m / 5 m	plants: flowers	air suction & rape	1.264	0.170	Inf	1.739	3.032E-01
0 m / 1 m	plants: stem & leaf	air suction & rape	2.656	0.360	Inf	7.213	3.304E-12
0 m / 3 m	plants: stem & leaf	air suction & rape	5.428	0.741	Inf	12.399	0.000E+00
0 m / 5 m	plants: stem & leaf	air suction & rape	6.862	0.934	Inf	14.155	0.000E+00
1 m / 3 m	plants: stem & leaf	air suction & rape	2.043	0.277	Inf	5.274	7.960E-07
1 m / 5 m	plants: stem & leaf	air suction & rape	2.583	0.348	Inf	7.043	1.133E-11
3 m / 5 m	plants: stem & leaf	air suction & rape	1.264	0.170	Inf	1.739	3.032E-01

Table S13. Post-hoc test results for the multi-sampler dataset (n = 1390 observations) showing contrasts between combinations of drilling method and adjacent crop (DMAC; SE = standard error, df = degrees of freedom). P values were adjusted using the Tukey method. Distance: distance from the edge of the drilling area.

contrast	sampler	distance [m]	ratio	SE	df	z.ratio	p.value
air pressure & mustard / air pressure & rape	Petri dish	0	1.642	0.988	Inf	0.824	6.883E-01
air pressure & mustard / air suction & rape	Petri dish	0	0.083	0.053	Inf	-3.910	2.715E-04
air pressure & rape / air suction & rape	Petri dish	0	0.051	0.030	Inf	-4.947	2.258E-06
air pressure & mustard / air pressure & rape	plants: flowers	0	1.215	0.735	Inf	0.322	9.445E-01
air pressure & mustard / air suction & rape	plants: flowers	0	0.042	0.027	Inf	-4.941	2.329E-06
air pressure & rape / air suction & rape	plants: flowers	0	0.034	0.021	Inf	-5.546	8.737E-08
air pressure & mustard / air pressure & rape	plants: stem & leaf	0	1.176	0.709	Inf	0.268	9.611E-01
air pressure & mustard / air suction & rape	plants: stem & leaf	0	0.081	0.052	Inf	-3.930	2.510E-04
air pressure & rape / air suction & rape	plants: stem & leaf	0	0.069	0.042	Inf	-4.410	3.082E-05
air pressure & mustard / air pressure & rape	Petri dish	1	1.887	1.136	Inf	1.054	5.427E-01
air pressure & mustard / air suction & rape	Petri dish	1	0.118	0.075	Inf	-3.355	2.286E-03
air pressure & rape / air suction & rape	Petri dish	1	0.062	0.038	Inf	-4.593	1.301E-05
air pressure & mustard / air pressure & rape	plants: flowers	1	1.396	0.844	Inf	0.552	8.454E-01
air pressure & mustard / air suction & rape	plants: flowers	1	0.059	0.038	Inf	-4.406	3.131E-05
air pressure & rape / air suction & rape	plants: flowers	1	0.042	0.026	Inf	-5.200	5.960E-07
air pressure & mustard / air pressure & rape	plants: stem & leaf	1	1.351	0.816	Inf	0.498	8.720E-01
air pressure & mustard / air suction & rape	plants: stem & leaf	1	0.115	0.073	Inf	-3.389	2.027E-03
air pressure & rape / air suction & rape	plants: stem & leaf	1	0.085	0.051	Inf	-4.071	1.388E-04
air pressure & mustard / air pressure & rape	Petri dish	3	2.199	1.322	Inf	1.311	3.891E-01
air pressure & mustard / air suction & rape	Petri dish	3	0.132	0.084	Inf	-3.181	4.186E-03
air pressure & rape / air suction & rape	Petri dish	3	0.060	0.036	Inf	-4.657	9.567E-06
air pressure & mustard / air pressure & rape	plants: flowers	3	1.628	0.982	Inf	0.807	6.986E-01
air pressure & mustard / air suction & rape	plants: flowers	3	0.066	0.042	Inf	-4.241	6.598E-05
air pressure & rape / air suction & rape	plants: flowers	3	0.041	0.025	Inf	-5.276	3.964E-07
air pressure & mustard / air pressure & rape	plants: stem & leaf	3	1.575	0.952	Inf	0.751	7.329E-01

Table S13 (continued).

contrast	sampler	distance [m]	ratio	SE	df	z.ratio	p.value
air pressure & mustard / air suction & rape	plants: stem & leaf	3	0.128	0.082	Inf	-3.204	3.872E-03
air pressure & rape / air suction & rape	plants: stem & leaf	3	0.081	0.049	Inf	-4.133	1.061E-04
air pressure & mustard / air pressure & rape	Petri dish	5	2.898	1.745	Inf	1.767	1.806E-01
air pressure & mustard / air suction & rape	Petri dish	5	0.169	0.108	Inf	-2.787	1.473E-02
air pressure & rape / air suction & rape	Petri dish	5	0.058	0.035	Inf	-4.699	7.818E-06
air pressure & mustard / air pressure & rape	plants: flowers	5	2.145	1.294	Inf	1.264	4.155E-01
air pressure & mustard / air suction & rape	plants: flowers	5	0.085	0.054	Inf	-3.854	3.420E-04
air pressure & rape / air suction & rape	plants: flowers	5	0.040	0.024	Inf	-5.322	3.069E-07
air pressure & mustard / air pressure & rape	plants: stem & leaf	5	2.075	1.256	Inf	1.206	4.494E-01
air pressure & mustard / air suction & rape	plants: stem & leaf	5	0.164	0.105	Inf	-2.816	1.350E-02
air pressure & rape / air suction & rape	plants: stem & leaf	5	0.079	0.048	Inf	-4.177	8.769E-05

Table S14. Post-hoc test results for the multi-sampler dataset (n = 1390 observations) showing contrasts between samplers (SE = standard error, df = degrees of freedom). P values were adjusted using the Tukey method. Distance: distance from the edge of the drilling area. DMAC = combinations of drilling method and adjacent crop.

contrast	distance [m]	DMAC	ratio	SE	df	z.ratio	p.value
Petri dish / plants: flowers	0	air pressure & mustard	1.046	0.112	Inf	0.420	9.075E-01
Petri dish / plants: stem & leaf	0	air pressure & mustard	0.533	0.056	Inf	-6.011	5.533E-09
plants: flowers / plants: stem & leaf	0	air pressure & mustard	0.510	0.059	Inf	-5.821	1.754E-08
Petri dish / plants: flowers	1	air pressure & mustard	1.046	0.112	Inf	0.420	9.075E-01
Petri dish / plants: stem & leaf	1	air pressure & mustard	0.533	0.056	Inf	-6.011	5.533E-09
plants: flowers / plants: stem & leaf	1	air pressure & mustard	0.510	0.059	Inf	-5.821	1.754E-08
Petri dish / plants: flowers	3	air pressure & mustard	1.046	0.112	Inf	0.420	9.075E-01
Petri dish / plants: stem & leaf	3	air pressure & mustard	0.533	0.056	Inf	-6.011	5.533E-09
plants: flowers / plants: stem & leaf	3	air pressure & mustard	0.510	0.059	Inf	-5.821	1.754E-08
Petri dish / plants: flowers	5	air pressure & mustard	1.046	0.112	Inf	0.420	9.075E-01
Petri dish / plants: stem & leaf	5	air pressure & mustard	0.533	0.056	Inf	-6.011	5.533E-09
plants: flowers / plants: stem & leaf	5	air pressure & mustard	0.510	0.059	Inf	-5.821	1.754E-08
Petri dish / plants: flowers	0	air pressure & rape	0.774	0.072	Inf	-2.765	1.572E-02
Petri dish / plants: stem & leaf	0	air pressure & rape	0.382	0.036	Inf	-10.187	2.742E-14
plants: flowers / plants: stem & leaf	0	air pressure & rape	0.493	0.048	Inf	-7.200	1.832E-12
Petri dish / plants: flowers	1	air pressure & rape	0.774	0.072	Inf	-2.765	1.572E-02
Petri dish / plants: stem & leaf	1	air pressure & rape	0.382	0.036	Inf	-10.187	2.742E-14
plants: flowers / plants: stem & leaf	1	air pressure & rape	0.493	0.048	Inf	-7.200	1.832E-12
Petri dish / plants: flowers	3	air pressure & rape	0.774	0.072	Inf	-2.765	1.572E-02
Petri dish / plants: stem & leaf	3	air pressure & rape	0.382	0.036	Inf	-10.187	2.742E-14
plants: flowers / plants: stem & leaf	3	air pressure & rape	0.493	0.048	Inf	-7.200	1.832E-12
Petri dish / plants: flowers	5	air pressure & rape	0.774	0.072	Inf	-2.765	1.572E-02
Petri dish / plants: stem & leaf	5	air pressure & rape	0.382	0.036	Inf	-10.187	2.742E-14
plants: flowers / plants: stem & leaf	5	air pressure & rape	0.493	0.048	Inf	-7.200	1.832E-12
Petri dish / plants: flowers	0	air suction & rape	0.527	0.075	Inf	-4.515	1.883E-05

Table S14 (continued).

contrast	distance [m]	DMAC	ratio	SE	df	z.ratio	p.value
Petri dish / plants: stem & leaf	0	air suction & rape	0.519	0.070	Inf	-4.848	3.723E-06
plants: flowers / plants: stem & leaf	0	air suction & rape	0.986	0.122	Inf	-0.113	9.930E-01
Petri dish / plants: flowers	1	air suction & rape	0.527	0.075	Inf	-4.515	1.883E-05
Petri dish / plants: stem & leaf	1	air suction & rape	0.519	0.070	Inf	-4.848	3.723E-06
plants: flowers / plants: stem & leaf	1	air suction & rape	0.986	0.122	Inf	-0.113	9.930E-01
Petri dish / plants: flowers	3	air suction & rape	0.527	0.075	Inf	-4.515	1.883E-05
Petri dish / plants: stem & leaf	3	air suction & rape	0.519	0.070	Inf	-4.848	3.723E-06
plants: flowers / plants: stem & leaf	3	air suction & rape	0.986	0.122	Inf	-0.113	9.930E-01
Petri dish / plants: flowers	5	air suction & rape	0.527	0.075	Inf	-4.515	1.883E-05
Petri dish / plants: stem & leaf	5	air suction & rape	0.519	0.070	Inf	-4.848	3.723E-06
plants: flowers / plants: stem & leaf	5	air suction & rape	0.986	0.122	Inf	-0.113	9.930E-01

Table S15. Post-hoc test results for the plant material-only dataset (n = 833 observations) showing contrasts between distances from the edge of the drilling area (SE = standard error, df = degrees of freedom). P values were adjusted using the Tukey method. DMAC: combination of drilling method and adjacent crop.

contrast	Sampler	DMAC	ratio	SE	df	z.ratio	p.value
0 m / 1 m	flowers	air pressure & mustard	2.188	0.279	Inf	6.139	4.968E-09
0 m / 3 m	flowers	air pressure & mustard	4.267	0.545	Inf	11.369	0.000E+00
0 m / 5 m	flowers	air pressure & mustard	4.337	0.559	Inf	11.374	0.000E+00
1 m / 3 m	flowers	air pressure & mustard	1.950	0.249	Inf	5.235	9.888E-07
1 m / 5 m	flowers	air pressure & mustard	1.983	0.256	Inf	5.304	6.768E-07
3 m / 5 m	flowers	air pressure & mustard	1.016	0.130	Inf	0.128	9.993E-01
0 m / 1 m	stem & leaf	air pressure & mustard	2.127	0.270	Inf	5.943	1.679E-08
0 m / 3 m	stem & leaf	air pressure & mustard	5.102	0.657	Inf	12.651	0.000E+00
0 m / 5 m	stem & leaf	air pressure & mustard	5.821	0.748	Inf	13.714	0.000E+00
1 m / 3 m	stem & leaf	air pressure & mustard	2.399	0.308	Inf	6.807	5.988E-11
1 m / 5 m	stem & leaf	air pressure & mustard	2.737	0.350	Inf	7.874	7.871E-14
3 m / 5 m	stem & leaf	air pressure & mustard	1.141	0.146	Inf	1.027	7.337E-01
0 m / 1 m	flowers	air pressure & rape	2.188	0.279	Inf	6.139	4.968E-09
0 m / 3 m	flowers	air pressure & rape	4.267	0.545	Inf	11.369	0.000E+00
0 m / 5 m	flowers	air pressure & rape	4.337	0.559	Inf	11.374	0.000E+00
1 m / 3 m	flowers	air pressure & rape	1.950	0.249	Inf	5.235	9.888E-07
1 m / 5 m	flowers	air pressure & rape	1.983	0.256	Inf	5.304	6.768E-07
3 m / 5 m	flowers	air pressure & rape	1.016	0.130	Inf	0.128	9.993E-01
0 m / 1 m	stem & leaf	air pressure & rape	2.127	0.270	Inf	5.943	1.679E-08
0 m / 3 m	stem & leaf	air pressure & rape	5.102	0.657	Inf	12.651	0.000E+00
0 m / 5 m	stem & leaf	air pressure & rape	5.821	0.748	Inf	13.714	0.000E+00
1 m / 3 m	stem & leaf	air pressure & rape	2.399	0.308	Inf	6.807	5.988E-11
1 m / 5 m	stem & leaf	air pressure & rape	2.737	0.350	Inf	7.874	7.871E-14
3 m / 5 m	stem & leaf	air pressure & rape	1.141	0.146	Inf	1.027	7.337E-01
0 m / 1 m	flowers	air suction & rape	2.188	0.279	Inf	6.139	4.968E-09
0 m / 3 m	flowers	air suction & rape	4.267	0.545	Inf	11.369	0.000E+00

Table S15 (continued).

contrast	Sampler	DMAC	ratio	SE	df	z.ratio	p.value
0 m / 5 m	flowers	air suction & rape	4.337	0.559	Inf	11.374	0.000E+00
1 m / 3 m	flowers	air suction & rape	1.950	0.249	Inf	5.235	9.888E-07
1 m / 5 m	flowers	air suction & rape	1.983	0.256	Inf	5.304	6.768E-07
3 m / 5 m	flowers	air suction & rape	1.016	0.130	Inf	0.128	9.993E-01
0 m / 1 m	stem & leaf	air suction & rape	2.127	0.270	Inf	5.943	1.679E-08
0 m / 3 m	stem & leaf	air suction & rape	5.102	0.657	Inf	12.651	0.000E+00
0 m / 5 m	stem & leaf	air suction & rape	5.821	0.748	Inf	13.714	0.000E+00
1 m / 3 m	stem & leaf	air suction & rape	2.399	0.308	Inf	6.807	5.988E-11
1 m / 5 m	stem & leaf	air suction & rape	2.737	0.350	Inf	7.874	7.871E-14
3 m / 5 m	stem & leaf	air suction & rape	1.141	0.146	Inf	1.027	7.337E-01

Table S16. Post-hoc test results for the plant material-only dataset (n = 833 observations) showing contrasts between combinations of drilling method and adjacent crop (DMAC; SE = standard error, df = degrees of freedom). P values were adjusted using the Tukey method. Distance: distance from the edge of the drilling area.

contrast	sampler	distance [m]	ratio	SE	df	z.ratio	p.value
air pressure & mustard / air pressure & rape	flowers	0	4.929	2.896	Inf	2.715	1.821E-02
air pressure & mustard / air suction & rape	flowers	0	0.327	0.218	Inf	-1.673	2.155E-01
air pressure & rape / air suction & rape	flowers	0	0.066	0.042	Inf	-4.256	6.173E-05
air pressure & mustard / air pressure & rape	stem & leaf	0	1.959	1.151	Inf	1.144	4.872E-01
air pressure & mustard / air suction & rape	stem & leaf	0	0.155	0.104	Inf	-2.788	1.467E-02
air pressure & rape / air suction & rape	stem & leaf	0	0.079	0.050	Inf	-3.977	2.057E-04
air pressure & mustard / air pressure & rape	flowers	1	4.929	2.896	Inf	2.715	1.821E-02
air pressure & mustard / air suction & rape	flowers	1	0.327	0.218	Inf	-1.673	2.155E-01
air pressure & rape / air suction & rape	flowers	1	0.066	0.042	Inf	-4.256	6.173E-05
air pressure & mustard / air pressure & rape	stem & leaf	1	1.959	1.151	Inf	1.144	4.872E-01
air pressure & mustard / air suction & rape	stem & leaf	1	0.155	0.104	Inf	-2.788	1.467E-02
air pressure & rape / air suction & rape	stem & leaf	1	0.079	0.050	Inf	-3.977	2.057E-04
air pressure & mustard / air pressure & rape	flowers	3	4.929	2.896	Inf	2.715	1.821E-02
air pressure & mustard / air suction & rape	flowers	3	0.327	0.218	Inf	-1.673	2.155E-01
air pressure & rape / air suction & rape	flowers	3	0.066	0.042	Inf	-4.256	6.173E-05
air pressure & mustard / air pressure & rape	stem & leaf	3	1.959	1.151	Inf	1.144	4.872E-01
air pressure & mustard / air suction & rape	stem & leaf	3	0.155	0.104	Inf	-2.788	1.467E-02
air pressure & rape / air suction & rape	stem & leaf	3	0.079	0.050	Inf	-3.977	2.057E-04
air pressure & mustard / air pressure & rape	flowers	5	4.929	2.896	Inf	2.715	1.821E-02
air pressure & mustard / air suction & rape	flowers	5	0.327	0.218	Inf	-1.673	2.155E-01
air pressure & rape / air suction & rape	flowers	5	0.066	0.042	Inf	-4.256	6.173E-05
air pressure & mustard / air pressure & rape	stem & leaf	5	1.959	1.151	Inf	1.144	4.872E-01
air pressure & mustard / air suction & rape	stem & leaf	5	0.155	0.104	Inf	-2.788	1.467E-02
air pressure & rape / air suction & rape	stem & leaf	5	0.079	0.050	Inf	-3.977	2.057E-04

Table S17. Post-hoc test results for the plant material-only dataset (n = 833 observations) showing contrasts between samplers (SE = standard error, df = degrees of freedom). P values were adjusted using the Tukey method. Distance: distance from the edge of the drilling area. DMAC = combinations of drilling method and adjacent crop.

contrast	distance [m]	DMAC	ratio	SE	df	z.ratio	p.value
flowers / stem & leaf	0	air pressure & mustard	8.049	1.309	Inf	12.819	1.284E-37
flowers / stem & leaf	1	air pressure & mustard	7.825	1.258	Inf	12.800	1.640E-37
flowers / stem & leaf	3	air pressure & mustard	9.625	1.593	Inf	13.683	1.283E-42
flowers / stem & leaf	5	air pressure & mustard	10.802	1.758	Inf	14.621	2.061E-48
flowers / stem & leaf	0	air pressure & rape	3.198	0.472	Inf	7.885	3.157E-15
flowers / stem & leaf	1	air pressure & rape	3.109	0.462	Inf	7.634	2.278E-14
flowers / stem & leaf	3	air pressure & rape	3.824	0.575	Inf	8.921	4.607E-19
flowers / stem & leaf	5	air pressure & rape	4.292	0.660	Inf	9.472	2.754E-21
flowers / stem & leaf	0	air suction & rape	3.819	0.635	Inf	8.053	8.062E-16
flowers / stem & leaf	1	air suction & rape	3.713	0.629	Inf	7.740	9.912E-15
flowers / stem & leaf	3	air suction & rape	4.567	0.764	Inf	9.076	1.128E-19
flowers / stem & leaf	5	air suction & rape	5.126	0.859	Inf	9.756	1.731E-22

Table S18. GLMM results for the Petri-dish-only dataset (n = 1218 observations), showing fitted mean values (fit; µg a.s./m²), standard error (se), as well as lower and upper 95% confidence limits (LCL and UCL, respectively) for the effect of the Heubach a.s. value (HVAS) [g active substance per ha] on measured residues.

HVAS	fit	se	LCL	UCL
6.755E-04	0.516	0.326	0.149	1.781
7.466E-04	0.534	0.328	0.160	1.784
8.251E-04	0.552	0.330	0.170	1.787
1.008E-03	0.590	0.334	0.194	1.793
1.114E-03	0.610	0.336	0.207	1.797
1.231E-03	0.631	0.337	0.221	1.801
1.360E-03	0.653	0.339	0.236	1.806
1.662E-03	0.698	0.340	0.268	1.818
1.836E-03	0.722	0.341	0.286	1.824
2.029E-03	0.746	0.341	0.304	1.831
2.243E-03	0.772	0.342	0.324	1.840
2.739E-03	0.825	0.341	0.367	1.859
3.028E-03	0.854	0.341	0.390	1.870
3.346E-03	0.883	0.341	0.414	1.882
4.087E-03	0.944	0.339	0.466	1.911
4.517E-03	0.976	0.339	0.494	1.928
4.992E-03	1.010	0.338	0.523	1.948
5.517E-03	1.044	0.338	0.554	1.969
6.738E-03	1.117	0.337	0.617	2.020
7.447E-03	1.155	0.338	0.650	2.050
8.230E-03	1.194	0.339	0.684	2.085
9.095E-03	1.235	0.341	0.718	2.123
1.111E-02	1.321	0.348	0.787	2.215
1.228E-02	1.366	0.354	0.822	2.270
1.357E-02	1.412	0.361	0.855	2.331
1.500E-02	1.460	0.370	0.889	2.400
1.832E-02	1.562	0.394	0.952	2.563
2.024E-02	1.615	0.410	0.981	2.659
2.237E-02	1.670	0.429	1.009	2.764
2.472E-02	1.727	0.450	1.035	2.881
3.020E-02	1.847	0.503	1.083	3.151
3.337E-02	1.910	0.534	1.104	3.305
3.688E-02	1.975	0.568	1.124	3.473
4.076E-02	2.043	0.606	1.142	3.656
4.979E-02	2.185	0.692	1.173	4.069
5.502E-02	2.259	0.741	1.187	4.301
6.081E-02	2.336	0.794	1.199	4.552
6.721E-02	2.416	0.851	1.211	4.822
8.208E-02	2.584	0.977	1.231	5.425
9.072E-02	2.672	1.046	1.239	5.762
